# Ethical Responsibility in Medical AI: A Semi-Systematic Thematic Review and Multilevel Governance Model

**DOI:** 10.3390/healthcare14030287

**Published:** 2026-01-23

**Authors:** Domingos Martinho, Pedro Sobreiro, Andreia Domingues, Filipa Martinho, Nuno Nogueira

**Affiliations:** 1 ISLA Santarém, Polytechnic University, Rua Dr. Teixeira Guedes, 31, 2000-029 Santarém, Portugal; andreia.domingues@islasantarem.pt (A.D.); filipa.martinho@islasantarem.pt (F.M.); nuno.nogueira@islasantarem.pt (N.N.); 2 NECE–UBI, Estrada do Sineiro 56, 6200-209 Covilhã, Portugal; 3 ESDRM, Polytechnic University of Santarém, 2001-904 Santarém, Portugal; sobreiro@esdrm.ipsantarem.pt

**Keywords:** algorithmic accountability, data protection and privacy, ethical responsibility, justice and equity, medical AI, patient autonomy, regulatory governance, transparency and explainability

## Abstract

**Highlights:**

**What are the main findings?**

- Transparency and explainability dominate ethical discourse (34.8%), whereas patient autonomy (8.6%) and professional redefinition (1.1%) are neglected.
- A fragmented accountability landscape emerges, with regulatory frameworks lagging behind technological innovation.

**What are the implications of the main findings?**

- Healthcare institutions must operationalise multilevel governance models that integrate ex ante (preventive) and ex post (accountability) mechanisms.
- Policymakers should mandate algorithmic audits and participatory design to bridge gaps in epistemic justice in AI-driven medicine.

**Abstract:**

**Background**: Artificial intelligence (AI) is transforming medical practice, enhancing diagnostic accuracy, personalisation, and clinical efficiency. However, this transition raises complex ethical challenges related to transparency, accountability, fairness, and human oversight. This study examines how the literature conceptualises and distributes ethical responsibility in AI-assisted healthcare. **Methods**: This semi-systematic, theory-informed thematic review was conducted in accordance with the PRISMA 2020 guidelines. Publications from 2020 to 2025 were retrieved from PubMed, ScienceDirect, IEEE Xplore databases, and MDPI journals. A semi-quantitative keyword-based scoring model was applied to titles and abstracts to determine their relevance. High-relevance studies (n = 187) were analysed using an eight-category ethical framework: transparency and explainability, regulatory challenges, accountability, justice and equity, patient autonomy, beneficence–non-maleficence, data privacy, and the impact on the medical profession. **Results**: The analysis revealed a fragmented ethical landscape in which technological innovation frequently outperforms regulatory harmonisation and shared accountability structures. Transparency and explainability were the dominant concerns (34.8%). Significant gaps in organisational responsibility, equitable data practices, patient autonomy, and professional redefinition were reported. A multilevel ethical responsibility model was developed, integrating micro (clinical), meso (institutional), and macro (regulatory) dimensions, articulated through both ex ante and ex post perspectives. **Conclusions**: AI requires governance frameworks that integrate ethical principles, regulatory alignment, and epistemic justice in medicine. This review proposes a multidimensional model that bridges normative ethics and operational governance. Future research should explore empirical, longitudinal, and interdisciplinary approaches to assess the real impact of AI on clinical practice, equity, and trust.

## 1. Introduction

Digital transformation has profoundly redefined the way health knowledge is produced, shared, and applied. Data-driven technologies, from predictive analytics to the Internet of Things, are reshaping the healthcare continuum, powering more personalised, proactive, and participatory medicine [1]. This transition is not only technological but also epistemological: clinical reasoning, which previously focused on the doctor’s individual experience, is now part of decision support systems based on algorithms and big data.

Artificial intelligence (AI), particularly machine learning and deep learning approaches, has emerged as one of the most disruptive drivers of this transformation. It is estimated that by 2030, more than 70% of healthcare organisations will integrate AI applications into their diagnostic, triage, and clinical management procedures [2]. Recent studies have shown gains in efficiency and diagnostic accuracy in multiple domains, from imaging to chronic disease screening, underpinning the promise of more efficient and equitable medicine [3]. Recent surveys indicate that less than 15% (14.8%) of physicians consider themselves to be “very familiar” with clinical AI tools, and almost half (47.2%) reported little or no familiarity with the available systems [4]. Estimates of hospitals that use algorithmic clinical decision support systems indicate that only 19% of institutions reported a high degree of success with regard to the use of diagnostic clinical AI [5].

However, the technical sophistication of these tools does not eliminate the accompanying ethical and epistemological uncertainties [6,7,8]. As the autonomy of systems increases, tensions emerge between innovation and precaution, efficiency and equity, clinical benefit, and protection of individual rights [9]. The risk of algorithmic opacity, often referred to as the “black box” problem, makes it difficult to explain and justify algorithms’ decisions [2,10,11]. Therefore, the transformative potential of AI in medicine requires reflection on the conditions under which the technology can be considered ethical, safe, and responsible [10,11,12].

This lack of explainability compromises not only the trust of professionals and patients but also the central principles of bioethics: autonomy, beneficence, non-maleficence, and justice [13]. An algorithm that recommends a therapy without the ability to provide an intelligible explanation challenges the principle of autonomy by limiting the patient’s informed consent and the physician’s ability to justify the decision [14]. However, the absence of transparency can lead to systematic errors that are not detectable, thereby questioning the principle of non-maleficence [15].

In addition, AI can reproduce and amplify inequalities. Models trained with biased data—for example, data obtained predominantly from Caucasian populations or from developed country contexts—tend to be less accurate when applied to ethnic minorities or under-represented populations [16]. Thus, distributive justice and equitable access to quality health care are threatened, contradicting one of the foundations of medical ethics and human rights [7,16].

The growing ambiguity in the attribution of responsibility is one of the most complex challenges identified in the literature. The nexus between action, intention, and consequence becomes diffuse when algorithms influence or determine clinical decisions [17]. This depersonalisation of medical decisions raises questions about who should be held accountable for errors, damages or omissions: the programmer, the institution, the doctor, or the system itself [18]?

The reviews published thus far address important aspects of AI ethics, such as transparency, algorithmic fairness, and data protection [19,20,21]. However, most of these studies maintain a normative or theoretical perspective, focusing on the abstract principles of bioethics (i.e., autonomy, beneficence, non-maleficence, and justice) without examining the institutional mechanisms of ethical governance or the operationalisation of responsibility in real contexts of clinical practice.

While the guidance on ethics and governance of AI presents for WHO [22] emphasises high-level principles and the European Union AI Act [23] focuses on regulatory compliance, our model uniquely integrates operational accountability mechanisms across clinical (micro), institutional (meso), and regulatory (macro) levels, articulated through ex ante (preventive) and ex post (responsive) dimensions.

Adopting AI-based solutions without in-depth ethical reflection can result in injustice, exclusion, and loss of trust in healthcare institutions. In this context, understanding the ethical dilemmas and boundaries of responsibility is an essential condition for ensuring that technological innovation remains subordinated to the principle of the primacy of the human being [24].

In view of this research gap, the main objective of this semi-systematic, theory-informed thematic review is to map and synthesise the ethical approaches to human responsibility in the application of AI to medicine, analysing how scientific studies conceptualise, operationalise, and propose mechanisms of ethical and institutional accountability. Specifically, this study seeks to answer the following question: How does the recent scientific literature address human responsibility in the use of AI in medical contexts and what are the ethical axes emerging from this reflection?

The analysis is structured around eight main categories: transparency and explainability, regulatory challenges, responsibility and accountability, justice and equity, patient autonomy, benign vs. non-maleficence, privacy and data protection, and impact on the medical profession, allowing us to understand the complexity and transversality of current ethical dilemmas.

## 2. Materials and Methods

This semi-systematic, theory-informed thematic review was conducted in accordance with the Preferred Reporting Items for Systematic Reviews and Meta-Analyses (PRISMA) 2020, which define international standards to ensure the transparency, reproducibility, and traceability of scientific reviews [25]. The protocol for this review was retrospectively registered on the Open Science Framework (ID: 10.17605/OSF.IO/DZUVT).

We adopted a semi-systematic, semi-quantitative review design, drawing on the mixed-methods approaches described by Snyder [25] and Paul and Criado [26], which seek to balance qualitative depth with transparent and reproducible procedures. In this context, an automated semantic keyword scoring model was used as an exploratory relevance filter, primarily to prioritise conceptually dense articles within a large initial corpus (n = 728), while acknowledging that this heuristic approach does not constitute a gold- standard selection method but rather a pragmatic aid to screening and comparability over time and themes [27,28,29,30,31]. This semi-systematic, theory-informed review operates as a synthesis strategy that provides the empirical and conceptual groundwork for the multilevel ethical responsibility model developed in this study, rather than as a formal hypothesis-testing design.

### 2.1. Selection Criteria and Research Strategy

This review is based on studies published between 2020 and 2025, and we collected scientific articles published in English. To maintain consistency in the interpretation of the data, articles published in languages other than English were excluded. In addition, studies outside the health domains, purely technical studies with no ethical component, non-academic opinion articles, studies without peer review, and duplicate works were excluded. The literature search was conducted between July and September 2025 in the following international databases: PubMed, ScienceDirect, and IEEE Xplore using Boolean combinations of descriptors in English (Table 1).

Additionally, we screened MDPI journals within the healthcare and biomedical portfolio as a targeted publisher search rather than as a bibliographic database. Boolean English descriptor combinations were used for screening (Table 2).

The literature search was conducted in PubMed, ScienceDirect and IEEE Xplore, complemented by a targeted search of MDPI journals within the healthcare and biomedical portfolio. MDPI was used as a targeted publisher search rather than as an independent bibliographic database, and ScienceDirect was consulted as an Elsevier platform, which systematically excludes journals from other major publishers. PubMed predominantly indexes biomedical literature, while IEEE Xplore is technically focused. As such, the search strategy prioritised clinically oriented and technical publications and should be interpreted as a focused synthesis rather than a fully comprehensive multidisciplinary search and is therefore best understood as a focused, semi-systematic mapping of clinically oriented literature rather than a fully comprehensive systematic review.

This combination of sources prioritised biomedical and technical journals and may therefore under-represent legal, philosophical and social science contributions to AI ethics in healthcare; as such, this review should be interpreted as a focused synthesis of clinically oriented literature rather than a fully comprehensive mapping of all disciplinary perspectives.

### 2.2. Review Process, Data Extraction, Analysis, and Classification

**Figure 1:**
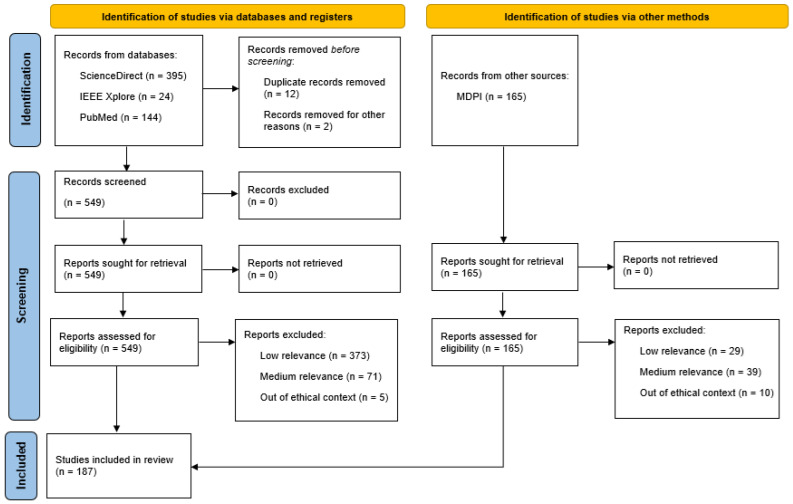
PRISMA 2020 flow diagram of the study selection process (n = 187 high-relevance studies included).

The data review and extraction process was conducted in three stages in accordance with PRISMA 2020 [24], as illustrated in Figure 1. Duplicate records were identified and removed in Zotero using automatic and manual procedures, and the numbers at each stage (initial records, duplicates removed, and final corpus) are reported in the PRISMA 2020 flow diagram to support transparency and reproducibility. Relevance was calculated using a semantic keyword scoring model, reflecting the intensity and centrality of each article’s ethical focus [32].

The coding and scoring of the articles were performed automatically using a semi-quantitative semantic score developed in Python 3.13 (pandas, regex/re, unidecode, spaCy). Keywords were detected in titles and abstracts [32] exported from Zotero.

This semantic keyword scoring model was used as a semi-quantitative, exploratory filter to prioritise conceptually dense articles within the large initial corpus, rather than as a perfect or definitive selection tool, with subsequent thematic interpretation complemented final inclusion decisions.

High-weight keywords (2 points each) represented explicit ethical concepts. Such keywords included “*ethic*,” “*bioethic*”, “*accountability*”, “*responsibility*”, “*liability*”, “*explainab*”, “*interpretab*”, “*xai*”, “*transparency*”, “*black box*”, “*autonomy*”, “*consent*”, “*informed consent*”, “*fairness*”, “*bias*”, “*equity*”, “*privacy*”, “*gdpr*”, “*data protection*”, “*trust*”, “*governance*”, and “*oversight*”. Medium-weight keywords (1 point each) represented the applied technical context of AI in medicine, and examples included “*AI in medicine*”, “*clinical AI*”, “*healthcare AI*”, “*decision support*” and “*medical AI*”.

To avoid additional selection bias, truncated forms, such as “*explainab*”, “*interpretab*” and “*xai*” were used exclusively in the internal semantic scoring model to capture variants (e.g., explainable, interpretability) and were not used as database search terms.

Keywords were weighted according to their conceptual centrality to ethical reasoning in AI-driven medicine to ensure a balanced yet discriminative classification. Terms explicitly representing ethical constructs were assigned a high weight (2 points), as they denote the normative and philosophical dimensions related to human responsibility. The terms referring to the applied or contextual domain of AI in healthcare received a medium weight (1 point) because they indicate disciplinary context but not necessarily ethical content. The final relevance score was obtained by summing the occurrences of both keyword categories in the titles and abstracts. Articles with a relevance score of six or more points were classified as highly relevant, resulting in a final corpus of 187 high-relevance studies.

It is important to distinguish between semantic relevance, which refers to the lexical density of ethical keywords in a text, and ethical pertinence, which expresses the conceptual depth with which an article addresses ethical responsibility. The first is measured automatically through keyword frequency and weighting; the second emerges through thematic interpretation of how those terms are framed within the article’s argument.

Potential sources of bias were identified and mitigated throughout the review process in accordance with PRISMA 2020 [24]. We sought to mitigate selection bias by applying uniform Boolean strings across databases, performing duplicate removal, and conducting dual screening on a stratified random subsample.

Two reviewers independently screened a 10% stratified random sample (n = 71) based on the title and abstract to validate the high-relevance classification (n = 714). Each article was rated (0 = low, 1 = medium, 2 = high). The inter-coder reliability, which was assessed using Cohen’s κ indicated almost perfect (k = 97%), supporting the robustness of the automated relevance filtering procedure [33].

### 2.3. Ethical Categories

The classification model adopted reflects the central concerns in international AI ethics frameworks in healthcare based on the weighted occurrence of keywords in titles and abstracts [7].

The quantitative distribution of ethical categories (Table 3) revealed a pronounced predominance of transparency and explainability (34.0%), regulatory challenges (19.0%), and responsibility and accountability (16.0%). This pattern confirms that the literature continues to prioritise the epistemic and regulatory foundations of trustworthy AI over micro-ethical or clinical dilemmas. In contrast, beneficence vs. non-maleficence (3.2%), impact on the medical profession (1.6%), and privacy and data protection (1.1%) remained marginal, indicating that practical and professional concerns remained secondary in the ethical debate (Table 3). It is important to emphasise that these frequencies indicate how prominently each ethical domain appears in the recent literature, rather than its intrinsic moral importance or priority in medical practice.

Supplementary File S4 contains a complete list of keywords organised by ethical category.

Although these ethical categories draw on broadly accepted bioethical and human rights-inspired principles, they should be understood as an interpretive framework rather than a universally fixed set of concepts. Their concrete meaning and implementation may vary across jurisdictions, reflecting differences in legal traditions, cultural norms, and institutional arrangements in healthcare and AI governance.

A validation procedure was conducted using a dual-coding approach to ensure the thematic classification reliability of the 187 included articles. A random sample of 30 articles (16% of the dataset) was independently coded by two reviewers. Inter-coder reliability was assessed using Cohen’s kappa, yielding κ = 0.80, which represents substantial agreement according to the Landis and Koch [33] scale. Consensus was used to resolve discrepancies, and the refined coding criteria were consistently applied to the entire corpus.

Bibliographic and contextual information (year of publication, region, and journal), clinical specialty, article type, and the main ethical issues addressed were extracted for each included study (n = 187). Each article was then assigned to one main ethical category from the eight-domain framework (transparency and explainability, regulatory challenges, responsibility and accountability, justice and equity, patient autonomy, beneficence vs. non-maleficence, privacy and data protection, and impact on the medical profession).

The evidence synthesis in this review is primarily narrative and conceptual: quantitative and descriptive claims about ethical themes, clinical domains, and geographical patterns are based on these extracted variables from the 187 included studies, whereas additional theoretical and regulatory sources are cited only for contextual and interpretive purposes and are not part of the systematic review corpus.

Because this review did not synthesise quantitative effect estimates but instead focused on conceptual and ethical dimensions, a formal risk-of-bias assessment (e.g., using ROB tools) was not undertaken. Potential bias was mitigated through duplicate screening, predefined eligibility criteria, and transparent reporting of the selection and classification processes.

No formal assessment of reporting bias or certainty of evidence (e.g., GRADE) was performed because the review did not aggregate quantitative effect sizes. Instead, we emphasised transparency in the search, selection, and classification procedures.

All 187 included studies are listed in the main reference list, and Supplementary File S1 contains their metadata and ethical classification.

### 2.4. Grey Literature Analysis

To complement the evidence obtained through the systematic review, a non-systematic scan of the grey literature was conducted to identify major regulatory and governance documents related to the ethical oversight of AI in healthcare (e.g., FDA [34], EMA [35], WHO [22]). This complementary analysis was not part of the PRISMA review process; did not contribute to study identification, screening, or eligibility; and did not inform the quantitative results. The purpose of this study was solely to support the interpretative comparison between academic ethical priorities and those expressed in international governance frameworks.

Only documents explicitly addressing ethics, governance, responsibility, liability, or risk management in medical or health-related AI were included; purely technical standards or non-health-specific documents were excluded.

This exploratory scan was methodologically distinct from and strictly separated from the PRISMA review; documents identified through this scan did not contribute to the study identification, screening, or eligibility, nor to the quantitative results. Supplementary File S5 contains a complete list of the relevant documents.

## 3. Results

Building on the descriptive results presented above, the analysis is deepened in the following subsections by unpacking the eight ethical domains that consistently appeared across the 187 high-relevance articles.

### 3.1. Geographical Origin

**Scheme 1:**
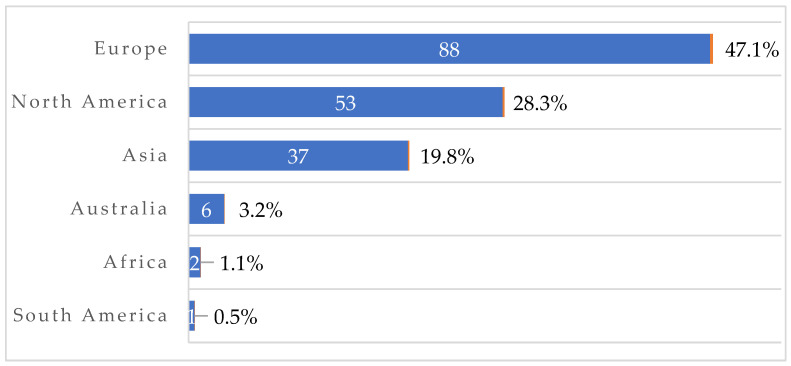
Geographic distribution of the included studies (n = 187).

The largest number of studies included in the review originated in Europe (47.1%), followed by North America (28.3%) and Asia (19.8%) (Scheme 1)

### 3.2. Temporal Distribution

**Scheme 2:**
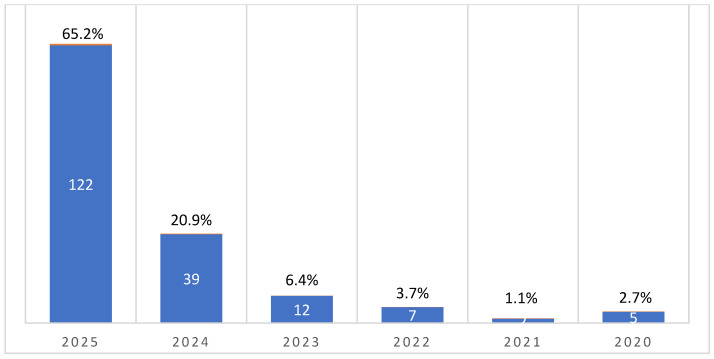
Temporal distribution of the included studies (n = 187).

Most of these studies were published in 2025 (65.2%), while about 20.9% were published in 2024 (Scheme 2).

### 3.3. Medical Areas/Specialties of Application

Of the 187 included studies, 128 were classified as clinical specialties. Likewise, the distribution of medical specialties (Table 4) demonstrates a concentration of studies in radiology and imaging (17.2%), public health (16.4%), and oncology (14.8%), reflecting the areas where algorithmic systems are most mature and where questions of transparency and responsibility have become the most visible. Together, these results underline the existence of an ethical asymmetry: research has deepened the epistemic and regulatory dimensions of AI in medicine while still under-representing its human - professional and distributive implications.

### 3.4. Ethical Issues and Dilemmas

The most relevant results related to ethical dilemmas are presented below, based on the eight categories identified, completing the analysis with the resulting impact/consequence for ethics and human responsibility. Consequently, the prominence of certain themes (e.g., transparency and regulation) in our results should be understood as a reflection of publication patterns, while other domains, such as autonomy or privacy, may be ethically central yet comparatively under-represented in the current evidence base.

For example, radiology and oncology studies often report tensions between diagnostic gains and the opacity of deep learning models used for image interpretation, whereas public health applications highlight concerns about algorithmic bias in triage and resource allocation [36,37,38]. Several tools designed for risk prediction or early warning illustrate how small errors in model calibration or data representativeness can have disproportionate consequences for vulnerable patient groups in cardiology and critical care settings [39,40].

#### 3.4.1. Transparency and Explainability

Transparency and explainability have emerged as central ethical dimensions associated with the application of AI in clinical contexts [36,41,42]. The growing complexity of algorithmic models, especially those based on *deep learning*, accentuates the “black box” character, making it difficult to understand the criteria underlying automated decisions [43,44,45,46,47,48,49,50,51,52,53,54,55,56,57].

Key concerns include (i) reduced model interpretability, (ii) inadequate explanations for clinicians, and (iii) non-auditable decision pathways, particularly in diagnostic and therapeutic contexts [58,59,60]. Among the proposed strategies, the adoption of explainable AI (XAI) approaches stands out, as they make inference processes intelligible, traceable, and verifiable through visual interpretation, multicentre validation, and algorithmic audits [15,36,41,45,49,50,51,52,54,56,57,61,62,63,64,65,66,67]. Several empirical and review studies show that XAI tools can improve clinicians’ perceived trust and acceptance of AI recommendations while also supporting more transparent patient communication [48,50,68,69,70,71,72,73,74,75,76,77,78].

#### 3.4.2. Regulatory Challenges

The articles in this category highlight the existence of regulatory and legal gaps in the regulation of AI in health applications [79,80,81,82,83,84,85,86,87,88,89,90]. Studies have pointed out that the speed of technological innovation has outpaced the pace of legislative reforms, leading to grey areas in the certification, validation, and accountability processes [91].

Specific challenges are mentioned with regard classifying software as a medical device (SaMD) or machine learning medical device (MLMD), given the dynamic nature of the algorithms and the absence of uniform standards for updating them [21,81,92,93,94,95,96,97].

The referenced studies also point to concerns about post-market monitoring and ongoing clinical validation, especially in devices that adapt with new data [59,98,99,100,101,102]. Adaptive and risk-based regulation models stand out among the proposed solutions, as they are capable of balancing innovation and security, reinforcing transparency and institutional trust [85,87,89,90,91,103,104,105,106,107,108].

Other contributions examine regulatory and professional-body guidance for AI-enabled telehealth and digital diagnostics, highlighting gaps between legal requirements and clinical practice [109,110,111,112,113,114].

#### 3.4.3. Responsibility and Accountability

Professional and institutional responsibility has emerged as one of the most recurrent ethical dilemmas in the application of AI to medicine affecting nurses, dentists, oncologists, and other professionals [108,115,116,117,118,119,120,121,122]. The analysed publications show a dispersion of responsibilities among various stakeholders, including developers, clinicians, health institutions, and regulatory entities, making it difficult to identify the responsible agent in case of error or clinical damage [18,115,123,124,125].

Shared responsibility models that articulate technical, ethical, and legal dimensions and advocate the need for continuous human oversight and transparent audit mechanisms have also emerged [17,20,37,85,87,88,126,127,128,129,130,131,132,133,134,135].

Some studies also emphasise the importance of institutional accountability structures and accountability matrices between human and non-human actors to ensure traceability and ethical security in the lifecycle of AI systems [123,136,137].

These recurring proposals for oversight structures, auditing mechanisms, and shared responsibility matrices directly inform the institutional and regulatory components of the multilevel governance model presented in Section 4.7.

The literature also proposes an inclusive and participatory ethics in AI system design, involving multidisciplinary teams and diverse communities in their validation, avoiding the perpetuation of racial gender or other inequalities [138,139].

#### 3.4.4. Justice and Equity

Studies in this category show that justice and equity are central dimensions in the ethical evaluation of AI applied to medicine [140,141,142,143,144,145,146,147]. When trained with historical and often non-representative data, clinical algorithms can reproduce and amplify structural inequalities that already exist in health systems [62,148,149,150,151,152]. Gender, age, and geographic origin biases that compromise equity of access and diagnostic accuracy in minority groups have been described in [39,149,153,154,155,156].

The development of fairness metrics to monitor the intergroup performance of algorithms and the implementation of regular ethical audits to ensure data representativeness stand out among the identified proposals [37,157,158,159,160,161].

The authors also emphasise the importance of participatory ethics, involving affected communities and experts from different areas in the design and validation of systems, ensuring a more inclusive and socially just approach to health innovation [62,162].

Recent work in digital health and nursing further illustrates how algorithmic tools can either mitigate or reinforce existing inequities, depending on data representativeness and deployment context [37,89,90,145,146,147,163,164].

#### 3.4.5. Patient Autonomy

Studies reveal that patient autonomy is undergoing transformation in the context of algorithmic medicine, and that this is taking place across domains such as dermatology, surgery, cardiology, and palliative care [77,165,166,167,168,169,170]. The introduction of AI systems capable of suggesting diagnoses, therapies, or prognostic decisions reduces the traditional space for shared decision-making, repositioning the role of the patient and the doctor in an ecosystem of technological co-decision [171,172,173,174,175,176].

The literature highlights concerns about the weakening of informed consent because many systems operate as “black boxes”, making it impossible to fully understand clinical recommendations [177,178,179]. Technical explainability should be accompanied by communicational transparency, allowing the patient to understand the limits, uncertainties, and potential biases of the algorithms [174,180].

Among the proposed solutions, digital health education, ethical and communicational training of professionals, and the co-construction of dynamic informed consent, adapted to the use of AI, stand out [181,182,183,184].

#### 3.4.6. Beneficence vs. Non-Maleficence

Our literature analysis demonstrates that the principle of beneficence, traditionally associated with the promotion of patient well-being, is reinterpreted in the context of algorithmic medicine in tension with the non-maleficence duty [75,185,186,187]. Although AI can improve diagnostic accuracy and clinical efficiency, its premature implementation or lack of adequate validation introduces new risks that must be mitigated, including classification errors, hidden biases, and overconfidence in the results generated [78,152,188,189].

Several authors have underlined the need for multi-centre and continuous evaluations before integrating AI systems into medical practice to ensure robustness, reliability, and patient safety [19]. Several studies have warned that the lack of explainability compromises the early detection of errors and can turn AI into a source of unintended harm, especially in contexts involving high clinical complexity [190].

#### 3.4.7. Impact on the Medical Profession

The introduction of AI is profoundly transforming the medical profession [106,191,192,193]. Currently, automated systems mediate diagnostic and clinical decision-making tasks, shifting the focus from technical skills to the functions of supervision, interpretation, and ethical validation [194].

The authors warn of the risk of progressive disqualification and technological dependence, which can compromise prudential reasoning and professional autonomy [115].

However, there is a need for new digital and ethical skills that are capable of sustaining a collaborative clinical practice between humans and algorithms [128,195].

#### 3.4.8. Privacy and Data Protection

Our literature analysis shows that one of the main challenges associated with integrating AI into clinical contexts is the ethical management of sensitive data. The referenced studies underline that AI systems rely on large volumes of biomedical and personal data, which carries risks of privacy violations, re-identification, and non-consensual secondary use [196,197,198].

The traditional mechanisms of anonymisation and pseudonymisation are insufficient in deep learning models, which can reconstruct individual profiles from anonymous data. The adoption of privacy principles integrated from the system design emerges as one of the main strategies to balance innovation with the protection of fundamental rights [81,103].

Some studies have highlighted the importance of transparency in data governance, arguing that the traceability of the information flow, from collection to analytical use, is essential to strengthen institutional trust and comply with the General Data Protection Regulation (GDPR) [199].

## 4. Discussion

This systematic review mapped the central ethical dimensions of human responsibility in medical AI and revealed a structurally fragmented landscape across clinical, organisational, and regulatory levels. Although the literature engages with eight core domains, transparency, regulation, accountability, justice, autonomy, beneficence, privacy, and professional transformation, these issues are often analysed separately, resulting in a diffusion of responsibility across the actors involved in AI-assisted clinical care. The following subsections synthesise these findings, explore theoretical contributions, and present practical and policy implications. The integrated conceptual model (Section 4.7) consolidates these insights into a unified multilevel framework.

### 4.1. Regulatory and Contextual Background

Major international governance frameworks for AI in healthcare, including the WHO [22] guidance on the ethics and governance of AI, emerging FDA [34] and EMA [35] initiatives on machine learning medical devices, and the EU AI Act [23], articulate overlapping concerns regarding risk-based classification, transparency, robustness, and lifecycle oversight, even if they differ in scope, legal status, and level of implementation. These documents provide a regulatory backdrop for interpreting our findings but do not form part of the PRISMA review corpus; they were identified through a separate, non-systematic scan and are used solely as contextual benchmarks in this Section 4.

Therefore, in this section of the paper, we treat them as a set of complementary soft-law and hard-law reference points rather than as a comprehensive mapping of all jurisdictional approaches to medical AI governance.

### 4.2. Synthesis of the Study Findings

The analysis reveals that medical AI has unevenly distributed ethical attention. Technical concerns, especially transparency and explainability, dominate the academic debate (34.8%), while other domains, such as privacy (1.1%) and autonomy (5.0%), receive limited systematic attention [49,50,51,52,54,56,57]. This focus reflects a tendency to prioritise algorithmic intelligibility and performance over relational, institutional, or systemic concerns [43,63,64]. Where justice and equity are addressed, studies tend to focus on bias in imaging, hepatology, and public health, as well as on proposals for fairness metrics and equity-oriented governance frameworks [73,75,76,144,150,155,170,200,201].

The analysis reveals that medical AI has unevenly distributed ethical attention. Technical concerns, especially transparency and explainability, dominate the academic debate (34.8%), while other domains, such as privacy (1.1%) and autonomy (5.0%), receive limited systematic attention. This focus reflects a tendency to prioritise algorithmic intelligibility and performance over relational, institutional, or systemic concerns. This predominance is reflected in numerous studies that propose explainable models, visualisation techniques, or interpretable decision-support tools in areas such as imaging, cardiology, and oncology [46,47,49,50,51,52,54,56,57,202,203]. At the same time, several studies in our corpus explicitly examine the experiences of patients and clinicians with AI-mediated care, including trust, perceived gains and losses in autonomy, and the reconfiguration of the therapeutic relationship, particularly in decision-support and emerging psychotherapeutic applications.

The inconsistent allocation of responsibility across the micro, meso, and macro levels is a second source of fragmentation. At the micro level, clinicians are positioned as the primary custodians of oversight; however, the increasing complexity of AI systems challenges their capacity to fully understand or contest algorithmic recommendations [48,74,77,168,169]. At the meso level, few healthcare institutions have established formal AI ethics committees, auditing routines, or incident-reporting mechanisms, revealing a gap between organisational responsibility and regulatory expectations [85,87,88,126,134,135,204]. At the macro level, regulatory bodies such as the FDA [34], EMA [35], WHO [22], and the EU AI Act [23] emphasise traceability, risk classification, and post-market surveillance, but these requirements are not yet operationalised within most healthcare systems and are discussed here as contextual regulatory benchmarks rather than as part of the systematically included study corpus [89,90,145,146,147].

These findings point to a diffusion of responsibility, where the ethical burden of AI-related decisions shifts unpredictably among developers, clinicians, institutions, and regulators [118,124,131,132]. This fragmentation underscores the need for structured, multilevel governance models that articulate clear accountability chains throughout the AI lifecycle.

The multilevel ethical responsibility model presented in Section 4.7 is derived from these thematic and descriptive patterns, translating observed gaps and proposals in the 187 studies into a structured micro–meso–macro governance architecture.

### 4.3. Academic Evidence and Regulatory Priorities

A structured comparison between academic evidence and regulatory guidance (Supplementary File S4) reveals substantial asymmetries in priorities. Academic literature emphasises transparency, explainability, epistemic justice, and the relational nature of clinical responsibility. In contrast, regulatory documents privilege risk classification, safety, accountability mechanisms, and lifecycle oversight. WHO [22] is the only major document to explicitly address global health equity, whereas instruments such as the FDA [34], EMA [35], and the EU AI Act [23] focus primarily on technical robustness and compliance. Several domain-specific frameworks in radiology, surgery, public health, and occupational health echo these concerns, proposing risk-stratified regulation, continuous performance monitoring, and context-sensitive safety standards [84,100,105,205]. These differences underline the need for integrated governance models capable of bridging ethical principles and regulatory implementation.

Notably, these regulatory and policy documents were identified through a separate, non-systematic grey literature scan and are used here solely for interpretive comparison, not as part of the PRISMA review corpus.

### 4.4. Theoretical Implications

This study contributes to the theoretical advancement of AI ethical governance in medicine by reconceptualising human responsibility as a multilevel and multidimensional construct.

The theoretical model proposed in this article emerges inductively from the structured synthesis of 187 high-relevance studies, organised into eight ethical domains. Thus, the systematic review operates as a bridge between dispersed empirical–conceptual evidence and the multilevel, ex ante/ex post accountability framework.

Rather than being understood only as attribution of blame after error, medical AI accountability should be viewed as a distributed process that unfolds over time, crossing different organisational levels and normative orientations [17,18,21,206].

The analysis sustains a temporal reformulation of responsibility, moving from reactive logic (ex post) to preventive logic (ex ante). The emphasis on design ethics, prior validation, and robust informed consent expands traditional bioethical principles into the algorithmic lifecycle, anticipating moral obligations rather than just sanctioning violations [8,82,207,208,209].

The distinction between micro (professional), meso (institutional), and macro (systemic) levels allows for the integration of discussions that have hitherto been fragmented between bioethics, health law, and organisational governance [79,92,94,206]. This approach demonstrates that ethical accountability in AI cannot be guaranteed only by doctors or programmers but results from interactions between professionals, institutions, and regulators.

The proposed accountability framework contributes theoretically by articulating three ethical traditions in a single analytical model. This pluralistic integration enables a more comprehensive understanding of how algorithms-mediated moral reasoning manifests itself in decisions, bringing normative ethics closer to clinical practice [9,45,60,63].

Finally, this study reinforces the conceptual link between explainability and responsibility. Transparency is not only a technical property but also an essential epistemic condition for moral and legal accountability [2,43,210,211], a point reinforced by applied XAI studies that explicitly connect interpretability with trust, consent quality, and oversight capacity in clinical settings [212,213]. This relationship provides a theoretical basis for assessing the influence of AI system design choices on human oversight and trust.

Together, these contributions extend existing ethical models in digital medicine, positioning liability as a dynamic, relational, and systemic concept, which can underpin new empirical and theoretical research on AI’s ethical and regulatory governance in health.

### 4.5. Practical and Policy Implications

This review highlights the urgent need to translate ethical principles into actionable mechanisms that ensure trustworthy, fair, and auditable AI applications in medicine. A multilevel governance structure is required to bridge the gap between normative ethics and clinical practice. This structure must be temporally aware and integrate preventive (ex ante) and corrective (ex post) accountability mechanisms distributed across the professional, institutional, and systemic domains.

From a research and development perspective, ethical oversight should begin at the design phase. This includes integrating explainability and transparency metrics into algorithmic validation processes, adopting multicentre evaluation methods, and ensuring data representativeness to avoid biased or discriminatory outcomes. Open documentation standards, such as dataset datasheets and model cards, can improve reproducibility and foster institutional trust by reducing informational asymmetry between developers, clinicians, and regulators.

At the clinical and educational levels, the responsible integration of AI into healthcare practice depends on the development of AI literacy and applied ethics competences. Clinicians must understand algorithmic reasoning, interpret limitations, and transparently communicate uncertainty to patients. Embedding ethical and digital literacy within medical and nursing curricula is essential for maintaining professional autonomy and ensuring meaningful human oversight. Continuous training in algorithmic interpretation and ethical risk management should be prioritised to prevent over-reliance on automation and to preserve moral agency in medical decision-making.

The findings underscore the importance of adaptive, evidence-based governance from institutional and regulatory perspectives. Healthcare institutions should establish AI Stewardship Committees or Ethics Oversight Boards responsible for predeployment evaluation, post-market surveillance, and ongoing bias monitoring. Regulators should move beyond static compliance models towards adaptive and risk-based regulation that can keep pace with the ML system’s evolution. This includes the implementation of continuous reporting systems for algorithmic incidents, independent audits, and ethical certification procedures for SaMD and MLMD.

Overall, the implications of this study reinforce that ethical AI governance is not a singular task but a distributed process involving shared responsibility across time and institutional boundaries. Table 5 summarises the proposed integrated framework of practical and policy actions according to the temporal dimension (ex ante/ex post) and levels of responsibility (micro, meso, or macro).

### 4.6. Contradictions and Gaps

This analysis reveals a set of internal contradictions that hinder the development of coherent ethical governance for medical AI [204]. The first point of tension is between explainability and privacy: achieving meaningful transparency often requires the exposition of sensitive features or decision pathways, which can conflict with GDPR-compliant anonymisation requirements [103].

A second contradiction arises in the relationship between AI bias and accountability. Although clinicians are formally expected to supervise algorithmic outputs, empirical evidence shows that they may over-rely on automated recommendations, thereby weakening the very human oversight that accountability frameworks presuppose [17,126].

The third tension concerns equity and performance optimisation. Many approaches to bias mitigation rely on technical adjustments to datasets or model parameters but pay limited attention to the structural determinants of health inequity, resulting in partial or superficial corrections [148,149,214]. A fourth contradiction appears between autonomy and workflow efficiency: while AI-driven optimisation can streamline decision processes, it may also reduce opportunities for clinician–patient dialogue, indirectly undermining relational autonomy even when formal consent mechanisms remain intact [171,172,173].

Finally, there is a persistent gap exists between regulatory expectations and institutional readiness. The EU AI Act [23], along with FDA [34] and EMA [35] guidance, imposes demanding requirements for traceability, auditing, and continuous monitoring. However, most healthcare organisations lack the governance infrastructure required to meet these obligations.

These contradictions highlight the difficulty of aligning technical performance with ethical legitimacy, institutional capacity, and human-centred care. They reinforce the need for integrated, multilevel governance models capable of reconciling these competing demands within a unified ethical and operational framework.

### 4.7. Integrated Conceptual Multilevel Ethical Responsibility Model for AI-Assisted Healthcare

**Figure 2:**
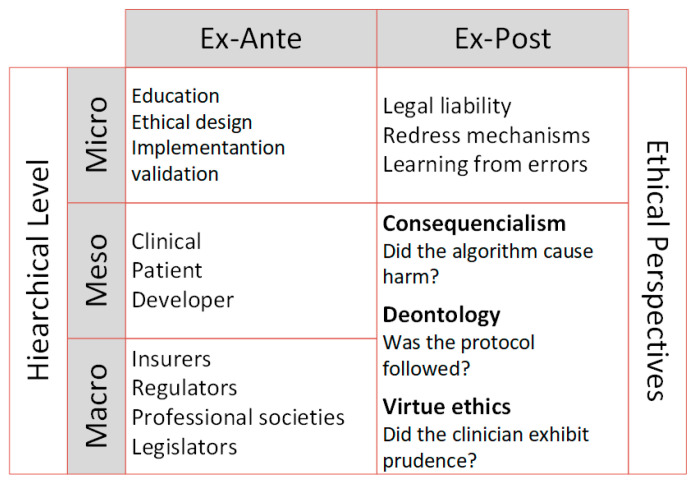
A multilevel ethical responsibility model for AI-assisted healthcare.

The integrated multilevel model of ethical responsibility in AI-assisted medicine that combines structural, temporal, and relational dimensions is shown in Figure 2.

Building on the thematic patterns identified in the eight ethical domains, the model primarily synthesises how the reviewed studies distribute responsibility across clinicians, organisations and regulators, while organising these insights into an integrated micro–meso–macro structure. The model synthesises insights from the literature on distributed responsibility in sociotechnical systems [6,8] and aligns them with the foundational principles of biomedical ethics (i.e., autonomy, beneficence, non-maleficence, and justice) [13]. Responsibility is structured across three interconnected levels (micro, meso, and macro) and articulated through two complementary temporal orientations: ex ante responsibility (before system deployment) and ex post responsibility (after clinical decisions and outcomes) [17]. Together, these elements illustrate how ethical responsibility is both distributed and dynamic in AI-enabled healthcare [204].

At the micro level, clinicians and other healthcare professionals are responsible for interpreting AI outputs, exercising clinical judgement, preserving informed consent, and safeguarding patients’ autonomy and dignity [171,172]. The potential for testimonial and hermeneutical injustices becomes salient in contexts where models operate as partial black boxes, reinforcing the need for meaningful explainability as a condition for ethically legitimate practice [64,215].

At the meso level, healthcare organisations, including hospitals, clinical governance boards, data-science teams, and risk-management units, play a central role in structuring accountability. This includes establishing validation protocols, monitoring performance drift, implementing fairness audits, and ensuring the safe integration of AI systems into clinical workflows [126]. This view aligns with organisational theories of responsibility that emphasise the importance of institutional routines, procedural safeguards, and auditability in complex healthcare environments [8,14,136].

At the macro level, regulators, policymakers, and technology developers assume system-level responsibility for setting standards, defining risk classifications, ensuring post-market surveillance, and promoting equity-driven data governance [81]. These roles reflect the broader ethical and societal expectations articulated in global AI governance frameworks, particularly regarding transparency, robustness, and fairness. In particular, developers are responsible for design choices, dataset representativeness, model interpretability, and mechanisms that support continuous oversight [43,149].

The model incorporates a transversal epistemic layer across all three levels, inspired by the notion of epistemic justice [215], emphasising the need for intelligible and equitable participation in AI-supported clinical decision-making [148,216,217]. This layer underscores that explainability is not only a technical requirement but a relational and moral obligation necessary for maintaining trust and preventing informational power asymmetries between clinicians, patients, institutions, and system developers [172,173].

The model provides a coherent ethical governance framework that bridges normative principles with operational practice by integrating structural levels (micro, meso, and macro), temporal axes (ex ante and ex post), and epistemic considerations [17,81].

Rather than proposing an entirely novel theoretical paradigm, the model offers a modest conceptual extension of existing frameworks by systematically linking micro-, meso-, and macro-level responsibilities to ex ante and ex post temporal orientations, thereby translating dispersed insights from the literature into a coherent governance architecture for medical AI.

It offers theoretical and practical foundations for strengthening accountability chains, guiding safer implementation, and supporting trustworthy AI deployment in healthcare systems [136].

To situate the proposed multilevel ethical responsibility model for AI-assisted healthcare within the broader landscape of AI ethics and governance, a comparative analysis was conducted against three influential frameworks: WHO [22], FDA [34], and the EU AI Act [23]. Table 6 summarises this comparison across three core dimensions: ex ante mechanisms, ex post accountability, and micro–meso–macro integration. 

Although existing frameworks offer essential ethical and regulatory foundations, they vary considerably in terms of operational clarity and multilevel coherence. WHO [22] articulates high-level ethical principles but provides minimal operational guidance for implementation. The FDA [34] advances practical recommendations on data quality, training, and human oversight, yet it does not establish a complete governance architecture across healthcare institutions. The EU AI Act [23] represents the most operationally detailed framework, defining explicit procedures for risk classification, conformity assessment, traceability, and post-market surveillance, although it still lacks a fully integrated micro–meso–macro perspective.

In contrast, the proposed multilevel ethical responsibility model (MER) provides high operational specificity by distributing preventive and reactive mechanisms across clinical, institutional, and regulatory actors. Its integration of structural, temporal, and epistemic layers addresses gaps in existing frameworks, offering a coherent governance approach capable of supporting trustworthy and accountable AI deployment across the entire lifecycle.

### 4.8. Limitations

This review has several limitations that should be considered when interpreting the findings. Publication bias may persist despite the comprehensive PRISMA search strategy, as studies with predominantly positive or technologically optimistic results are more likely to be published.

The review protocol was not prospectively registered, which may increase the risk of unreported deviations from the initial plan. Relying on a semi-quantitative, automated semantic scoring model as an initial exploratory filter is a key methodological limitation. Although this approach improves the transparency and replicability of the screening process, it cannot be considered a gold-standard selection method and may misclassify conceptually rich studies that use alternative terminology.

The heterogeneity of study designs, clinical domains, and conceptual frameworks limits the ability to perform direct comparisons across studies. This variability reflects the diversity of the field but constrains the development of metrics for unified ethical responsibility. The analysis is based only on published literature; grey literature, policy documents, and institutional guidelines were not systematically included, potentially excluding emerging governance practices.

The rapid evolution of AI technologies means that ethical challenges may change faster than the literature can capture. Despite these limitations, this review provides a robust synthesis of the current ethical landscape and highlights critical gaps that warrant further empirical and policy-oriented investigation.

The keyword-based semantic scoring model used as an exploratory filter may misclassify conceptually rich studies that employ alternative terminology for ethical issues, particularly in philosophical and legal traditions (e.g., deontological duty rather than responsibility). Therefore, it should be regarded as a potential source of selection bias rather than a solution to it. Future studies should complement this type of heuristic, keyword-based approach with concept-based ontologies or other advanced semantic methods to better capture equivalent concepts across disciplines and reduce the risk of under-representing philosophical and legal inquiry.

In addition, the choice of information sources introduces additional constraints. The combination of PubMed, ScienceDirect, IEEE Xplore, and targeted MDPI journal searches prioritised biomedical and technical literature and did not include broader multidisciplinary databases (such as Scopus or Web of Science) or systematic searches of grey literature repositories. This may have introduced biases related to publisher and discipline, under-representing contributions from legal, philosophical and social science traditions and limiting the corpus’s completeness and representativeness. Therefore, future reviews should expand the database coverage and incorporate structured grey literature searches to better capture these perspectives.

Taken together, these methodological choices mean that the present work should be interpreted as a focused, semi-systematic thematic review of clinically oriented literature, rather than a fully comprehensive, unbiased systematic review.

## 5. Conclusions

This review proposes the first operationalizable multilevel model of ethical responsibility in medical AI, distinguishing preventive (ex ante) and reactive (ex post) mechanisms across clinical, institutional, and regulatory levels. The framework addresses the fragmentation of responsibility identified in the literature by integrating eight ethical domains with structural, temporal, and epistemic layers and provides a coherent foundation for the implementation of trustworthy and accountable AI in healthcare settings.

Based on the findings, concrete recommendations emerge. Hospitals should establish AI Oversight Committees with the authority to evaluate fairness, monitor performance drift, and audit algorithmic decisions throughout the system’s lifecycle. Regulatory bodies and developers should jointly require explainability-by-design in all high-risk AI systems to ensure that clinicians and patients can understand and contest algorithmic outputs. Professional organisations and academic institutions should integrate AI literacy and applied ethics into medical and technical curricula to enable clinicians to interpret model limitations, communicate uncertainty, and maintain moral agency in hybrid decision environments.

Future research should explore some underdeveloped avenues based on the collected evidence. Longitudinal empirical studies are needed to determine whether the adoption of explainable AI improves patient trust, quality of informed consent, and therapeutic adherence.

In addition, organisational research should also investigate how different institutional governance structures, such as AI Stewardship Committees or AI-IR systems, shape accountability in practice.

Interdisciplinary studies should examine the interaction of algorithmic transparency, fairness interventions, and regulatory obligations in real clinical settings, particularly in under-resourced or structurally inequitable health systems.

A systematic comparison of how these ethical categories are interpreted and operationalised in different national contexts was beyond the scope of this review, but it represents a crucial direction for future research on medical AI ethics and regulation.

Future research should apply the proposed multilevel responsibility framework to specific telemedicine scenarios and remote diagnostic tools (e.g., computer-vision systems for abdominal assessment), which could not be examined in depth within the scope of this broad ethical-governance review.

Together, these contributions reinforce the need for a coordinated and ethically grounded approach to AI deployment in medicine, ensuring that technological innovation strengthens rather than undermines the principles of human dignity, justice, and clinical responsibility.

## Figures and Tables

**Table 1:** Search strings.

Database/Journals	Search String	Fields	Filters
PubMed	(“artificial intelligence” OR “machine learning” OR “deep learning”) AND (“medicine” OR “clinical” OR “healthcare”) AND (“ethics” OR “responsibility” OR “accountability” OR “liability”)	Title/Abstract	2020–2025
ScienceDirect	“artificial intelligence” AND (“medicine” OR “clinical” OR “healthcare”) AND (“ethics” OR “responsibility” OR “accountability” OR “liability”)	Title/Abstract	2020–2025, Relevance (first 395)
IEEE Xplore	(“artificial intelligence” OR “machine learning” OR “deep learning”) AND (“medicine” OR “clinical” OR “healthcare”) AND (“ethics” OR “responsibility” OR “accountability” OR “liability”)	Title/Abstract	2020–2025, *Journal of Artificial Intelligence in Healthcare*

**Table 2:** Search strings.

Publisher	Search String	Fields	Filters
MDPI	(“artificial intelligence” OR “machine learning” OR “deep learning”) AND (“medicine” OR “clinical” OR “healthcare”) AND (“ethics” OR “responsibility” OR “accountability” OR “liability”)	Title/Abstract	2020–2025, journals (*Diagnostics*, *Cancers*, *Healthcare*, *Medicine*, *Biomedicines*, *Pharmaceuticals*, *Biomedinformatics*, *Biomolecules*, *Imaging*)

**Table 3:** Ethical categories.

Categories	Number	Percentage (%)
Transparency and Explainability	65	34.0%
Regulatory Challenges	36	19.0%
Responsibility and Accountability	30	16.0%
Justice and Equity	29	15.5%
Patient Autonomy	16	8.6%
Beneficence vs. Non-Maleficence	6	3.2%
Impact on the Medical Profession	3	1.6%
Privacy and Data Protection	2	1.1%

**Table 4:** Clinical specialty could be identified in 128 of the 187 high-relevance studies; frequencies and percentages in this table refer to this subset (n = 128).

Clinical Specialty	No. of Items	Percentage (%)
Radiology/Imaging	22	17.2%
Public health	21	16.4%
Oncology	19	14.8%
Cardiology	16	12.5%
Psychiatry/Mental Health	14	10.9%
Dentistry	7	5.5%
Paediatrics	6	4.7%
Surgery	6	4.70%
Nursing	4	3.1%
Neurology	3	2.3%
Internal/General Medicine	3	2.3%
Pharmacy/Pharmacology	2	1.6%
Geriatrics	2	1.6%
Ophthalmology	1	0.8%
Rehabilitation/Physiotherapy	1	0.8%
Dermatology	1	0.8%
	128	

**Table 5:** Implications for practice and policy.

Temporal Dimension	Level of Action	Ethical Focus	Policy and Practice Recommendations
Ex ante (preventive)	Micro (individual: clinician, patient, developer)	Transparency; autonomy; beneficence	Integrate AI literacy and applied ethics into medical and technical education.
			Clinicians must demonstrate a basic understanding of the functioning, interpretability, and limitations of AI models.
			Dynamic informed consent procedures to enable patients to update their data preferences and receive transparent explanations.
	Meso (institutional: hospitals, developers, insurers)	Accountability; Justice; data protection	Establish AI ethics oversight committees within healthcare institutions to assess AI fairness and patient impact before deployment.
			Mandate algorithmic audits and explainability checks before clinical implementation.
			Adopt institutional policies that ensure continuous bias monitoring and validation after deployment.
	Macro (systemic: regulators, professional bodies, legislators)	Regulatory adaptation; fairness	Aligning national legislation with the European Union AI Act and WHO guidance on ethical AI in health.
			Develop regulatory sandboxes to allow supervised testing of innovative AI systems in clinical environments.
			Introduce certification standards that explicitly include ethical compliance criteria for SaMD.
Ex post (reactive)	Micro (individual: clinician, patient, developer)	Accountability; non-maleficence	Define clear reporting pathways for AI-related clinical incidents and promote a “just culture” of learning rather than punishment.
			Promote professional codes of conduct that include responsibility for algorithmic supervision and decision override.
	Meso (institutional: hospitals, developers, insurers)	Transparency; responsibility	Implement traceability systems (decision logs, model versioning) to enable the retrospective auditing of algorithmic decisions.
			Create AI Stewardship Committees tasked with postimplementation review, safety monitoring, and clinical governance policy updating.
	Macro (systemic: regulators, professional bodies, legislators	Regulatory oversight; public trust	Establish national databases for adverse events related to medical AI, such as pharmacovigilance systems.
			Funding for independent ethical and social impact audits of medical AI technologies.
			Legal protection for whistleblowers reporting the unapproved or unsafe use of AI in healthcare.

**Table 6:** Comparison of existing frameworks with the proposed multilevel ethical responsibility model.

Dimension	WHO [22]	FDA [34]	EU AI Act [23]	MER
Operational guidance	Low operational specificity	Medium operationalisation	High operationalisation	High operationalisation
Ex ante mechanisms	Implicit guidance	Partial but operational focus	Explicit requirements for high-risk AI	Detailed preventive mechanisms
Ex post accountability	No structured mechanisms	Limited ex- post structure	Legal liability	Integrated clinical + organisational + legal
Micro–meso–macro integration	No multilevel specification	No explicit multilevel structure	Partial integration	Full multilevel integration

## Data Availability

No new data were created or analyzed in this study.

## Supplementary Materials

The following supporting information can be downloaded at https://www.mdpi.com/article/10.3390/healthcare14030287/s1, File S1: Supplementary File S1 contains a complete list of the included studies (n = 187), including title, publication year, journal, primary ethical theme, region, clinical specialty, article type, and DOI. File S2: Python script for relevance scoring. File S3: Python script for ethical categorisation. File S4: Complete keyword list by ethical category. File S5: Lists regulatory and governance documents identified through a non-systematic grey literature scan, used solely for interpretive comparison with the academic corpus and not as part of the PRISMA review.
