# Supplementary material for: Ethical Responsibility in Medical AI: A Semi-Systematic Thematic Review and Multilevel Governance Model
Source: Healthcare (Basel). 2026 Jan 23;14(3):287. doi: 10.3390/healthcare14030287 (PMC12897712; doi:10.3390/healthcare14030287)
Supplement: Supplementary file 1 [file healthcare-14-00287-s001.zip › healthcare-4035250-supplementary.pdf]

Supplementary Materials

File S1: List of included studies (n = 187) with bibliographic metadata.

ID	Title	Year	Journal	Main Ethical Theme	Region	Clinical specialty	Article type	DOI
1	A Conceptual Framework for Applying Ethical Principles of AI to Medical Practice	2025	Bioengineering	Transparency and Explainability	North America	Public health	Narrative Review / Perspective	10.3390/bioengineering12020180
2	A Privacy-Preserving and Attack-Aware AI Approach for High-Risk Healthcare Systems Under the EU AI Act	2025	Electronics	Regulatory Challenges	Europe	Not specified	Journal Article	10.3390/electronics14071385
3	A Research Landscape of Agentic AI and Large Language Models: Applications, Challenges and Future Directions	2025	Algorithms	Responsibility and Accountability	Europe	Not specified	Scoping Review	10.3390/a18080499
4	A State-of-the-Art Review of Artificial Intelligence (AI) Applications in Healthcare: Advances in Diabetes, Cancer, Epidemiology, and Mortality Prediction	2025	Computers	Transparency and Explainability	North America	Public health	Journal Article	10.3390/computers14040143
5	A systematic literature review of health consumer attitudes towards secondary use and sharing of health administrative and clinical trial data: a focus on privacy, trust, and transparency	2020	Systematic Reviews	Transparency and Explainability	Australia	Not specified	Systematic Review	10.1186/s13643-020-01481-9
6	Adversarial Examples on XAI-Enabled DT for Smart Healthcare Systems	2024	Sensors	Transparency and Explainability	Asia	Radiology / Imaging	Narrative Review / Perspective	10.3390/s24216891
7	Agentic LLM-based robotic systems for real-world applications: a review on their agenticness and ethics	2025	Frontiers in Robotics and AI	Regulatory Challenges	Europe	Not specified	Narrative Review / Perspective	10.3389/frobt.2025.1605405
8	AI and machine learning ethics, law, diversity, and global impact	2023	The British Journal of Radiology	Regulatory Challenges	North America	Oncology	Journal Article	10.1259/bjr.20220934
9	AI and Machine Learning in Transplantation	2025	Transplantology	Transparency and Explainability	Europe	Not specified	Journal Article	10.3390/transplantology6030023
10	AI in Biomedicine—A Forward-Looking Perspective on Health Equity	2024	International Journal of Environmental Research and Public Health	Justice and Equity	North America	Not specified	Narrative Review / Perspective	10.3390/ijerph21121642
11	AI in Dentistry: Innovations, Ethical Considerations, and Integration Barriers	2025	Bioengineering	Transparency and Explainability	Asia	Dentistry	Narrative Review / Perspective	10.3390/bioengineering12090928

ID	Title	Year	Journal	Main Ethical Theme	Region	Clinical specialty	Article type	DOI
12	AI-Driven Technology in Heart Failure Detection and Diagnosis: A Review of the Advancement in Personalized Healthcare	2025	Symmetry	Transparency and Explainability	North America	Cardiology	Journal Article	10.3390/sym17030469
13	AI-Driven Telerehabilitation: Benefits and Challenges of a Transformative Healthcare Approach	2025	AI	Justice and Equity	Europe	Rehabilitation/Physiotherapy	Journal Article	10.3390/ai6030062
14	AI-Driven Wearable Bioelectronics in Digital Healthcare	2025	Biosensors	Regulatory Challenges	North America	Cardiology	Journal Article	10.3390/bios15070410
15	AI-Powered Object Detection in Radiology: Current Models, Challenges, and Future Direction	2025	Journal of Imaging	Transparency and Explainability	Asia	Radiology / Imaging	Journal Article	10.3390/jimaging11050141
16	Algorethics in Healthcare: Balancing Innovation and Integrity in AI Development	2024	Algorithms	Responsibility and Accountability	Europe	Not specified	Narrative Review / Perspective	10.3390/a17100432
17	Applications of Generative Artificial Intelligence in Electronic Medical Records: A Scoping Review	2025	Information	Transparency and Explainability	North America	Not specified	Systematic Review	10.3390/info16040284
18	Applications of Machine Learning Algorithms in Geriatrics	2025	Applied Sciences	Transparency and Explainability	Europe	Geriatrics	Systematic Review	10.3390/app15158699
19	Applying Smart Healthcare and ESG Concepts to Optimize Elderly Health Management	2025	Sustainability	Responsibility and Accountability	Asia	Geriatrics	Journal Article	10.3390/su17136091
20	Artificial Intelligence (AI) and Emergency Medicine: Balancing Opportunities and Challenges	2025	JMIR Medical Informatics	Transparency and Explainability	Europe	Public health	Narrative Review / Perspective	10.2196/70903
21	Artificial Intelligence and Assistive Robotics in Healthcare Services: Applications in Silver Care	2025	International Journal of Environmental Research and Public Health	Regulatory Challenges	Europe	Public health	Narrative Review / Perspective	10.3390/ijerph22050781
22	Artificial Intelligence and Decision-Making in Oncology: A Review of Ethical, Legal, and Informed Consent Challenges	2025	Current Oncology Reports	Patient Autonomy	Europe	Oncology	Systematic Review	10.1007/s11912-025-01698-8
23	Artificial intelligence and smile design: An e-Delphi consensus statement of ethical challenges	2024	Journal of Prosthodontics	Responsibility and Accountability	Europe	Dentistry	Journal Article	10.1111/jopr.13858
24	Artificial Intelligence and the Future of Mental Health in a Digitally Transformed World	2025	Computers	Responsibility and Accountability	Europe	Psychiatry/Mental Health	Systematic Review	10.3390/computers14070259

ID	Title	Year	Journal	Main Ethical Theme	Region	Clinical specialty	Article type	DOI
25	Artificial intelligence at the pen's edge: Exploring the ethical quagmires in using artificial intelligence models like ChatGPT for assisted writing in biomedical research	2024	Perspectives in Clinical Research	Transparency and Explainability	Asia	Not specified	Narrative Review / Perspective	10.4103/picr.picr_196_23
26	Artificial intelligence for good health: a scoping review of the ethics literature	2021	BMC Medical Ethics	Justice and Equity	Europe	Public health	Scoping Review	10.1186/s12910-021-00577-8
27	Artificial Intelligence in Clinical Medicine: Challenges Across Diagnostic Imaging, Clinical Decision Support, Surgery, Pathology, and Drug Discovery	2025	Clinics and Practice	Justice and Equity	Europe	Radiology / Imaging	Journal Article	10.3390/clinpract15090169
28	Artificial intelligence in colorectal multidisciplinary team meetings. What are the medicolegal implications?	2024	Colorectal Disease	Patient Autonomy	Europe	Oncology	Journal Article	10.1111/codi.17091
29	Artificial Intelligence in Dental Education: Opportunities and Challenges of Large Language Models and Multimodal Foundation Models	2024	JMIR Medical Education	Transparency and Explainability	North America	Dentistry	Journal Article	10.2196/52346
30	Artificial intelligence in healthcare and medicine: clinical applications, therapeutic advances, and future perspectives	2025	European Journal of Medical Research	Transparency and Explainability	Africa	Public health	Journal Article	10.1186/s40001-025-03196-w
31	Artificial Intelligence in Healthcare: How to Develop and Implement Safe, Ethical and Trustworthy AI Systems	2025	AI	Responsibility and Accountability	North America	Not specified	Journal Article	10.3390/ai6060116
32	Artificial Intelligence in Healthcare: University Students' Perceptions and Level of Confidence	2025	Healthcare	Responsibility and Accountability	Europe	Public health	Journal Article	10.3390/healthcare13182312
33	Artificial Intelligence in Predictive Healthcare: A Systematic Review	2025	Journal of Clinical Medicine	Transparency and Explainability	Asia	Radiology / Imaging	Systematic Review	10.3390/jcm14196752
34	Artificial Intelligence in Primary Care: Support or Additional Burden on Physicians' Healthcare Work?—A Qualitative Study	2025	Clinics and Practice	Impact on the Medical Profession	Europe	Internal / General Medicine	Narrative Review / Perspective	10.3390/clinpract15080138
35	Artificial Intelligence in Primary Malignant Bone Tumor Imaging: A Narrative Review	2025	Diagnostics	Transparency and Explainability	North America	Oncology	Narrative Review / Perspective	10.3390/diagnostics15131714
36	Artificial Intelligence's Role in Improving Adverse Pregnancy Outcomes: A Scoping Review and Consideration of Ethical Issues	2025	Journal of Clinical Medicine	Justice and Equity	Europe	Radiology / Imaging	Systematic Review	10.3390/jcm14113860

ID	Title	Year	Journal	Main Ethical Theme	Region	Clinical specialty	Article type	DOI
37	Artificial Intelligence-Based Software as a Medical Device (AI-SaMD): A Systematic Review	2025	Healthcare	Responsibility and Accountability	Asia	Radiology / Imaging	Systematic Review	10.3390/healthcare13070817
38	Artificial Intelligence-Guided Neuromodulation in Heart Failure with Preserved and Reduced Ejection Fraction: Mechanisms, Evidence, and Future Directions	2025	Journal of Cardiovascular Development and Disease	Justice and Equity	North America	Cardiology	Journal Article	10.3390/jcdd12080314
39	Assessing Clinicians' Legal Concerns and the Need for a Regulatory Framework for AI in Healthcare: A Mixed-Methods Study	2025	Healthcare	Regulatory Challenges	Asia	Not specified	Narrative Review / Perspective	10.3390/healthcare13131487
40	Assessing Medical Students' Perceptions of AI-Integrated Telemedicine: A Cross-Sectional Study in Romania	2025	Healthcare	Responsibility and Accountability	Europa	Not specified	Quantitative / Observational Study	10.3390/healthcare13090990
41	Assessing the Impact of New Technologies on Managing Chronic Respiratory Diseases	2024	Journal of Clinical Medicine	Transparency and Explainability	Europe	Not specified	Journal Article	10.3390/jcm13226913
42	Attribution-Based Explainability in Medical Imaging: A Critical Review on Explainable Computer Vision (X-CV) Techniques and Their Applications in Medical AI	2025	Electronics	Transparency and Explainability	Europe	Radiology / Imaging	Journal Article	10.3390/electronics14153024
43	Balancing Privacy and Progress: A Review of Privacy Challenges, Systemic Oversight, and Patient Perceptions in AI-Driven Healthcare	2024	Applied Sciences	Patient Autonomy	North America	Not specified	Journal Article	10.3390/app14020675
44	Balancing Technology, Ethics, and Society: A Review of Artificial Intelligence in Embryo Selection	2025	Information	Justice and Equity	Europe	Not specified	Narrative Review / Perspective	10.3390/info16010018
45	Beyond Black Boxes: Interpretable AI with Explainable Neural Networks (ENNs) for Acute Myocardial Infarction (AMI) Using Common Hematological Parameters	2025	Medicina	Transparency and Explainability	Europe	Cardiology	Journal Article	10.3390/medicina61091552
46	Beyond Global Metrics: The U-Smile Method for Explainable, Interpretable, and Transparent Variable Selection in Risk Prediction Models	2025	Applied Sciences	Transparency and Explainability	Europe	Public health	Journal Article	10.3390/app15158303
47	Beyond Post hoc Explanations: A Comprehensive Framework for Accountable AI in Medical Imaging Through Transparency, Interpretability, and Explainability	2025	Bioengineering	Transparency and Explainability	North America	Radiology / Imaging	Systematic Review	10.3390/bioengineering12080879
48	Bias Mitigation via Synthetic Data Generation: A Review	2024	Electronics	Justice and Equity	Europe	Not specified	Journal Article	10.3390/electronics13193909

ID	Title	Year	Journal	Main Ethical Theme	Region	Clinical specialty	Article type	DOI
49	Biases in AI: acknowledging and addressing the inevitable ethical issues	2025	Frontiers in Digital Health	Regulatory Challenges	Europe	Psychiatry/Mental Health	Journal Article	10.3389/fdgth.2025.1614105
50	Bioethical Considerations of Deploying Artificial Intelligence in Clinical Orthopedic Settings: A Narrative Review	2025	HSS Journal®: The Musculoskeletal Journal of Hospital for Special Surgery	Justice and Equity	North America	Surgery	Systematic Review	10.1177/15563316251340303
51	Black box no more: a scoping review of AI governance frameworks to guide procurement and adoption of AI in medical imaging and radiotherapy in the UK	2023	The British Journal of Radiology	Transparency and Explainability	Europe	Oncology	Scoping Review	10.1259/bjr.20221157
52	Bridging Domains: Advances in Explainable, Automated, and Privacy-Preserving AI for Computer Science and Cybersecurity	2025	Computers	Transparency and Explainability	North America	Not specified	Narrative Review / Perspective	10.3390/computers14090374
53	Bridging the Gap: From AI Success in Clinical Trials to Real-World Healthcare Implementation—A Narrative Review	2025	Healthcare	Responsibility and Accountability	Asia	Not specified	Narrative Review / Perspective	10.3390/healthcare13070701
54	Bug Wars: Artificial Intelligence Strikes Back in Sepsis Management	2025	Diagnostics	Transparency and Explainability	Europe	Oncology	Systematic Review	10.3390/diagnostics15151890
55	Challenges of Artificial Intelligence in Medicine	2025	Studies in Health Technology and Informatics	Beneficence vs Non-Maleficence	Asia	Not specified	CHAP	10.3233/SHTI250039
56	ChatGPT and Digital Transformation: A Narrative Review of Its Role in Health, Education, and the Economy	2025	Digital	Justice and Equity	North America	Psychiatry/Mental Health	Narrative Review / Perspective	10.3390/digital5030024
57	ChatGPT and the Future of Digital Health: A Study on Healthcare Workers' Perceptions and Expectations	2023	Healthcare	Responsibility and Accountability	Asia	Public health	Quantitative / Observational Study	10.3390/healthcare11131812
58	Clearing the Fog: A Scoping Literature Review on the Ethical Issues Surrounding Artificial Intelligence-Based Medical Devices	2024	Journal of Personalized Medicine	Regulatory Challenges	Europe	Not specified	Scoping Review	10.3390/jpm14050443
59	Clinical Needs Assessment of a Machine Learning–Based Asthma Management Tool: User-Centered Design Approach	2024	JMIR Formative Research	Transparency and Explainability	North America	Pediatrics	Narrative Review / Perspective	10.2196/45391

ID	Title	Year	Journal	Main Ethical Theme	Region	Clinical specialty	Article type	DOI
60	Cost, Usability, Credibility, Fairness, Accountability, Transparency, and Explainability Framework for Safe and Effective Large Language Models in Medical Education: Narrative Review and Qualitative Study	2024	JMIR AI	Regulatory Challenges	Asia	Not specified	Narrative Review / Perspective	10.2196/51834
61	Current Trends and Future Directions of Digital Pathology and Artificial Intelligence in Dermatopathology: A Scientometric-Based Review	2025	Diagnostics	Regulatory Challenges	Europe	Oncology	Narrative Review / Perspective	10.3390/diagnostics15172196
62	Dentistry in the Era of Artificial Intelligence: Medical Behavior and Clinical Responsibility	2025	Prosthesis	Patient Autonomy	Europe	Dentistry	Systematic Review	10.3390/prosthesis7040095
63	Developing a Behavioral Phenotyping Layer for Artificial Intelligence–Driven Predictive Analytics in a Digital Resiliency Course: Protocol for a Randomized Controlled Trial	2025	JMIR Research Protocols	Regulatory Challenges	North America	Psychiatry/Mental Health	Narrative Review / Perspective	10.2196/73773
64	Development and Feasibility Study of HOPE Model for Prediction of Depression Among Older Adults Using Wi-Fi-based Motion Sensor Data: Machine Learning Study	2025	JMIR Aging	Transparency and Explainability	North America	Psychiatry/Mental Health	Quantitative / Observational Study	10.2196/67715
65	Digital Convergence in Dental Informatics: A Structured Narrative Review of Artificial Intelligence, Internet of Things, Digital Twins, and Large Language Models with Security, Privacy, and Ethical Perspectives	2025	Electronics	Regulatory Challenges	North America	Dentistry	Narrative Review / Perspective	10.3390/electronics14163278
66	Economic, ethical, and regulatory dimensions of artificial intelligence in healthcare: an integrative review	2025	Frontiers in Public Health	Regulatory Challenges	Asia	Public health	Systematic Review	10.3389/fpubh.2025.1617138
67	Efficacy of an Artificial Intelligence App (Aysa) in Dermatological Diagnosis: Cross-Sectional Analysis	2024	JMIR Dermatology	Patient Autonomy	Asia	Psychiatry/Mental Health	Quantitative / Observational Study	10.2196/48811
68	Engaging an advisory board in discussions about the ethical relevance of algorithmic bias and fairness	2025	npj Digital Medicine	Justice and Equity	North America	Not specified	Journal Article	10.1038/s41746-025-01711-1
69	Enhancing Ophthalmic Diagnosis and Treatment with Artificial Intelligence	2025	Medicina	Responsibility and Accountability	Europe	Ophthalmology	Journal Article	10.3390/medicina61030433
70	Era of Generalist Conversational Artificial Intelligence to Support Public Health Communications	2025	Journal of Medical Internet Research	Regulatory Challenges	North America	Public health	Narrative Review / Perspective	10.2196/69007

ID	Title	Year	Journal	Main Ethical Theme	Region	Clinical specialty	Article type	DOI
71	Establishing Best Practices for Clinical GWAS: Tackling Imputation and Data Quality Challenges	2025	International Journal of Molecular Sciences	Justice and Equity	North America	Public health	Narrative Review / Perspective	10.3390/ijms26136397
72	Ethical and legal considerations in healthcare AI: innovation and policy for safe and fair use	2025	Royal Society Open Science	Regulatory Challenges	Europe	Surgery	Journal Article	10.1098/rsos.241873
73	Ethical and regulatory considerations in the use of AI and machine learning in nursing: A systematic review	2025	International Nursing Review	Responsibility and Accountability	Asia	Nursing	Systematic Review	10.1111/inr.70010
74	Ethical and Social Implications of Using Predictive Modeling for Alzheimer's Disease Prevention: A Systematic Literature Review	2020	Journal of Alzheimer's Disease	Beneficence vs Non-Maleficence	Europe	Neurology	Qualitative Study	10.3233/JAD-191159
75	Ethical aspects of artificial intelligence: what urologists need to know	2025	Current Opinion in Urology	Transparency and Explainability	Europe	Surgery	Journal Article	10.1097/MOU.0000000000001278
76	Ethical considerations for artificial intelligence in dermatology: a scoping review	2024	British Journal of Dermatology	Patient Autonomy	North America	Dermatology	Systematic Review	10.1093/bjd/ljae040
77	Ethical considerations in telehealth and artificial intelligence for work related musculoskeletal disorders: A scoping review	2024	Work	Regulatory Challenges	Europe	Pharmacy / Pharmacology	Scoping Review	10.3233/WOR-240187
78	Ethical decision-making and artificial intelligence in nursing education: An integrative review	2025	Nursing Ethics	Justice and Equity	Europe	Nursing	Journal Article	10.1177/09697330251366600
79	Ethical Design of Data-Driven Decision Support Tools for Improving Cancer Care: Embedded Ethics Review of the 4D PICTURE Project	2025	JMIR Cancer	Justice and Equity	Europe	Oncology	Narrative Review / Perspective	10.2196/65566
80	Ethical framework for responsible foundational models in medical imaging	2025	Frontiers in Medicine	Transparency and Explainability	North America	Radiology / Imaging	Narrative Review / Perspective	10.3389/fmed.2025.1544501
81	Ethical Guidelines for Direct-To-Consumer Promotion of Transcranial Magnetic Stimulation	2025	American Journal of Therapeutics	Patient Autonomy	Europe	Psychiatry/Mental Health	Journal Article	10.1097/MJT.0000000000001974
82	Ethical implications of AI and robotics in healthcare: A review	2023	Medicine	Regulatory Challenges	North America	Not specified	Journal Article	10.1097/MD.0000000000036671
83	Ethical Implications of Artificial Intelligence in Vaccine Equity: Protocol for Exploring Vaccine Distribution Planning and Scheduling in Pandemics in Low- and Middle-Income Countries	2025	JMIR Research Protocols	Justice and Equity	Asia	Not specified	Systematic Review	10.2196/76634

ID	Title	Year	Journal	Main Ethical Theme	Region	Clinical specialty	Article type	DOI
84	Ethical Implications of Chatbot Utilization in Nephrology	2023	Journal of Personalized Medicine	Privacy and Data Protection	North America	Not specified	Journal Article	10.3390/jpm13091363
85	Ethical Principles in Digital Palliative Care for Children: The MyPal Project and Experiences Made in Designing a Trustworthy Approach	2022	Frontiers in Digital Health	Regulatory Challenges	Europe	Pediatrics	Narrative Review / Perspective	10.3389/fdgth.2022.730430
86	Ethical theories, governance models, and strategic frameworks for responsible AI adoption and organizational success	2025	Frontiers in Artificial Intelligence	Responsibility and Accountability	Europe	Not specified	Journal Article	10.3389/frai.2025.1619029
87	Ethics of artificial intelligence in embryo assessment: mapping the terrain	2025	Human Reproduction	Transparency and Explainability	Australia	Pediatrics	Journal Article	10.1093/humrep/deae264
88	Ethics of Blockchain by Design: Guiding a Responsible Future for Healthcare Innovation	2024	Blockchain in Healthcare Today	Responsibility and Accountability	Europe	Cardiology	Narrative Review / Perspective	10.30953/bhty.v7.362
89	Ethics of Wearable-Based Out-of-Hospital Cardiac Arrest Detection	2024	Circulation: Arrhythmia and Electrophysiology	Patient Autonomy	Europe	Cardiology	Journal Article	10.1161/CIRCEP.124.012913
90	Ethics, Bias, and Governance in Artificial Intelligence for Hepatology: Toward Building a Safe and Fair Future	2025	Journal of Clinical and Experimental Hepatology	Justice and Equity	Asia	Not specified	Journal Article	10.1016/j.jceh.2025.102628
91	Evaluating Trustworthiness in AI: Risks, Metrics, and Applications Across Industries	2025	Electronics	Regulatory Challenges	Europe	Not specified	Narrative Review / Perspective	10.3390/electronics14132717
92	Explainable AI (xAI) for Anatomic Pathology	2020	Advances in Anatomic Pathology	Transparency and Explainability	North America	Not specified	Journal Article	10.1097/PAP.0000000000000264
93	Explainable AI for Bioinformatics: Methods, Tools and Applications	2023	Briefings in Bioinformatics	Transparency and Explainability	Europe	Oncology	Journal Article	10.1093/bib/bbad236
94	Explainable AI in Clinical Decision Support Systems: A Meta-Analysis of Methods, Applications, and Usability Challenges	2025	Healthcare	Transparency and Explainability	Asia	Oncology	Systematic Review	10.3390/healthcare13172154
95	Explainable artificial intelligence (XAI) in radiology and nuclear medicine: a literature review	2023	Frontiers in Medicine	Transparency and Explainability	Europe	Radiology / Imaging	Journal Article	10.3389/fmed.2023.1180773
96	Explainable artificial intelligence for mental health through transparency and interpretability for understandability	2023	npj Digital Medicine	Transparency and Explainability	Europe	Psychiatry/Mental Health	Narrative Review / Perspective	10.1038/s41746-023-00751-9

ID	Title	Year	Journal	Main Ethical Theme	Region	Clinical specialty	Article type	DOI
97	Explainable Artificial Intelligence in Radiological Cardiovascular Imaging—A Systematic Review	2025	Diagnostics	Transparency and Explainability	Europe	Cardiology	Systematic Review	10.3390/diagnostics15111399
98	Explainable Artificial Intelligence Recommendation System by Leveraging the Semantics of Adverse Childhood Experiences: Proof-of-Concept Prototype Development	2020	JMIR Medical Informatics	Transparency and Explainability	North America	Pediatrics	Narrative Review / Perspective	10.2196/18752
99	Explainable Boosting Machines Identify Key Metabolomic Biomarkers in Rheumatoid Arthritis	2025	Medicina	Transparency and Explainability	Asia	Cardiology	Journal Article	10.3390/medicina61050833
100	Explainable, trustworthy, and ethical machine learning for healthcare: A survey	2022	Computers in Biology and Medicine	Transparency and Explainability	Asia	Not specified	Quantitative / Observational Study	10.1016/j.compbiomed.2022.106043
101	Exploring Artificial Intelligence Biases in Predictive Models for Cancer Diagnosis	2025	Cancers	Responsibility and Accountability	North America	Oncology	Journal Article	10.3390/cancers17030407
102	Exploring Opportunities and Challenges of AI in Primary Healthcare: A Qualitative Study with Family Doctors in Lithuania	2025	Healthcare	Responsibility and Accountability	Europe	Oncology	Qualitative Study	10.3390/healthcare13121429
103	Exploring the Applications of Explainability in Wearable Data Analytics: Systematic Literature Review	2024	Journal of Medical Internet Research	Transparency and Explainability	Asia	Not specified	Journal Article	10.2196/53863
104	Exploring the bioethical implications of using artificial intelligence in writing research proposals	2024	Perspectives in Clinical Research	Responsibility and Accountability	Asia	Not specified	Journal Article	10.4103/picr.picr_226_23
105	Exploring the Ethical Challenges of Conversational AI in Mental Health Care: Scoping Review	2025	JMIR Mental Health	Beneficence vs Non-Maleficence	Europe	Psychiatry/Mental Health	Scoping Review	10.2196/60432
106	Exploring the Potential of Digital Twins in Cancer Treatment: A Narrative Review of Reviews	2025	Journal of Clinical Medicine	Regulatory Challenges	Europe	Oncology	Narrative Review / Perspective	10.3390/jcm14103574
107	Fairness of artificial intelligence in healthcare: review and recommendations	2024	Japanese Journal of Radiology	Justice and Equity	Asia	Radiology / Imaging	Journal Article	10.1007/s11604-023-01474-3
108	From black box to clarity: Strategies for effective AI informed consent in healthcare	2025	Artificial Intelligence in Medicine	Transparency and Explainability	Australia	Not specified	Journal Article	10.1016/j.artmed.2025.103169

ID	Title	Year	Journal	Main Ethical Theme	Region	Clinical specialty	Article type	DOI
109	From Innovation to Regulation: Insights from a Bibliometric Analysis of Research Patterns in Medical Data Governance	2025	Informatics	Responsibility and Accountability	Europe	Not specified	Journal Article	10.3390/informatics12030066
110	Future Use of AI in Diagnostic Medicine: 2-Wave Cross-Sectional Survey Study	2025	Journal of Medical Internet Research	Regulatory Challenges	South America	Cardiology	Quantitative / Observational Study	10.2196/53892
111	Gaps in the Global Regulatory Frameworks for the Use of Artificial Intelligence (AI) in the Healthcare Services Sector and Key Recommendations	2024	Healthcare	Regulatory Challenges	Asia	Not specified	Journal Article	10.3390/healthcare12171730
112	Generative Artificial Intelligence in Healthcare: Applications, Implementation Challenges, and Future Directions	2025	BioMedInformatics	Responsibility and Accountability	Asia	Radiology / Imaging	Journal Article	10.3390/biomedinformatics5030037
113	Generative Artificial Intelligence Tools in Medical Research (GAMER): Protocol for a Scoping Review and Development of Reporting Guidelines	2025	JMIR Research Protocols	Transparency and Explainability	Asia	Not specified	Scoping Review	10.2196/64640
114	Governing Artificial Intelligence in Radiology: A Systematic Review of Ethical, Legal, and Regulatory Frameworks	2025	Diagnostics	Responsibility and Accountability	Asia	Radiology / Imaging	Systematic Review	10.3390/diagnostics15182300
115	Governing Data and Artificial Intelligence for Health Care: Developing an International Understanding	2022	JMIR Formative Research	Regulatory Challenges	Europe	Not specified	Scoping Review	10.2196/31623
116	Harnessing Artificial Intelligence in Pediatric Oncology Diagnosis and Treatment: A Review	2025	Cancers	Transparency and Explainability	North America	Oncology	Journal Article	10.3390/cancers17111828
117	Harnessing the power of synthetic data in healthcare: innovation, application, and privacy	2023	npj Digital Medicine	Responsibility and Accountability	North America	Public health	Journal Article	10.1038/s41746-023-00927-3
118	Healthcare experiences of pregnant and postnatal women and healthcare professionals when facing child protection in the perinatal period: A systematic review and Critical Interpretative Synthesis	2024	PLOS ONE	Regulatory Challenges	Europe	Pediatrics	Systematic Review	10.1371/journal.pone.0305738
119	How Could Artificial Intelligence Change the Doctor–Patient Relationship? A Medical Ethics Perspective	2025	Healthcare	Patient Autonomy	Europe	Radiology / Imaging	Narrative Review / Perspective	10.3390/healthcare13182340
120	Impact of Responsible AI on the Occurrence and Resolution of Ethical Issues: Protocol for a Scoping Review	2024	JMIR Research Protocols	Regulatory Challenges	North America	Not specified	Systematic Review	10.2196/52349

ID	Title	Year	Journal	Main Ethical Theme	Region	Clinical specialty	Article type	DOI
121	Informed consent and bioethical advances in clinical settings	2025	Frontiers in Psychology	Patient Autonomy	Europe	Psychiatry/Mental Health	Narrative Review / Perspective	10.3389/fpsyg.2025.1654586
122	Integrating AI and Assistive Technologies in Healthcare: Insights from a Narrative Review of Reviews	2025	Healthcare	Regulatory Challenges	Europa	Pediatrics	Narrative Review / Perspective	10.3390/healthcare13050556
123	Integrating Artificial Intelligence into Perinatal Care Pathways: A Scoping Review of Reviews of Applications, Outcomes, and Equity	2025	International Journal of Mental Health Nursing	Justice and Equity	Asia	Cardiology	Narrative Review / Perspective	10.3390/nursrep15080281
124	Integrating Artificial Intelligence With Workforce Solutions for Sustainable Care: A Follow Up to Artificial Intelligence and Machine Learning Based Decision Support Systems in Mental Health	2025	Nursing Reports	Transparency and Explainability	Australia	Psychiatry/Mental Health	Scoping Review	10.1111/inm.70019
125	Integrating Explainable Machine Learning in Clinical Decision Support Systems: Study Involving a Modified Design Thinking Approach	2024	JMIR Formative Research	Transparency and Explainability	North America	Radiology / Imaging	Narrative Review / Perspective	10.2196/50475
126	Integrating health equity in artificial intelligence for public health in Canada: a rapid narrative review	2025	Frontiers in Public Health	Transparency and Explainability	North America	Public health	Narrative Review / Perspective	10.3389/fpubh.2025.1524616
127	Integrating Large Language Models into Medication Management in Remote Healthcare: Current Applications, Challenges, and Future Prospects	2025	Systems	Regulatory Challenges	Asia	Pharmacy / Pharmacology	Journal Article	10.3390/systems13040281
128	Interpretable Prediction of Myocardial Infarction Using Explainable Boosting Machines: A Biomarker-Based Machine Learning Approach	2025	Diagnostics	Transparency and Explainability	Europe	Cardiology	Journal Article	10.3390/diagnostics15172219
129	Knowledge Graphs and Their Reciprocal Relationship with Large Language Models	2025	Machine Learning and Knowledge Extraction	Transparency and Explainability	North America	Not specified	Journal Article	10.3390/make7020038
130	Laboratory medicine between technological innovation, rights safeguarding, and patient safety: A bioethical perspective	2025	Open Medicine	Patient Autonomy	Europe	Not specified	Narrative Review / Perspective	10.1515/med-2025-1153
131	Large language models and generative AI in telehealth: a responsible use lens	2024	Journal of the American Medical Informatics Association	Transparency and Explainability	Australia	Not specified	Scoping Review	10.1093/jamia/ocae035

ID	Title	Year	Journal	Main Ethical Theme	Region	Clinical specialty	Article type	DOI
132	Large Language Models in Medical Chatbots: Opportunities, Challenges, and the Need to Address AI Risks	2025	Information	Responsibility and Accountability	North America	Psychiatry/Mental Health	Journal Article	10.3390/info16070549
133	Legal and Ethical Consideration in Artificial Intelligence in Healthcare: Who Takes Responsibility?	2022	Frontiers in Surgery	Regulatory Challenges	Asia	Cardiology	Journal Article	10.3389/fsurg.2022.862322
134	Leveraging Artificial Intelligence to Optimize Transcranial Direct Current Stimulation for Long COVID Management: A Forward-Looking Perspective	2024	Brain Sciences	Justice and Equity	Europe	Psychiatry/Mental Health	Narrative Review / Perspective	10.3390/brainsci14080831
135	Machine Learning and Artificial Intelligence in Intensive Care Medicine: Critical Recalibrations from Rule-Based Systems to Frontier Models	2025	Journal of Clinical Medicine	Transparency and Explainability	Europe	Cardiology	Journal Article	10.3390/jcm14124026
136	Mapping Ethical Guidelines for AI in Healthcare: A Global Perspective	2025	Studies in Health Technology and Informatics	Justice and Equity	Europe	Not specified	CHAP	10.3233/SHTI250624
137	Mitigating Ethical Issues for Large Language Models in Oncology: A Systematic Review	2025	JCO Clinical Cancer Informatics	Responsibility and Accountability	North America	Oncology	Systematic Review	10.1200/CCI-25-00076
138	Multimodal AI in Biomedicine: Pioneering the Future of Biomaterials, Diagnostics, and Personalized Healthcare	2025	Nanomaterials	Regulatory Challenges	Asia	Radiology / Imaging	Journal Article	10.3390/nano15120895
139	On the practical, ethical, and legal necessity of clinical Artificial Intelligence explainability: an examination of key arguments	2025	BMC Medical Informatics and Decision Making	Patient Autonomy	North America	Not specified	Journal Article	10.1186/s12911-025-02891-2
140	Operationalizing and Implementing Pretrained, Large Artificial Intelligence Linguistic Models in the US Health Care System: Outlook of Generative Pretrained Transformer 3 (GPT-3) as a Service Model	2022	JMIR Medical Informatics	Transparency and Explainability	North America	Not specified	Narrative Review / Perspective	10.2196/32875
141	Patient Perspectives on Artificial Intelligence in Medical Imaging	2025	Journal of Participatory Medicine	Impact on the Medical Profession	North America	Radiology / Imaging	Narrative Review / Perspective	10.2196/67816
142	Patients' Perceptions Toward Human–Artificial Intelligence Interaction in Health Care: Experimental Study	2021	Journal of Medical Internet Research	Beneficence vs Non-Maleficence	North America	Psychiatry/Mental Health	Quantitative / Observational Study	10.2196/25856

ID	Title	Year	Journal	Main Ethical Theme	Region	Clinical specialty	Article type	DOI
143	Personalized Medical Approach in Gastrointestinal Surgical Oncology: Current Trends and Future Perspectives	2025	Journal of Personalized Medicine	Transparency and Explainability	Asia	Oncology	Journal Article	10.3390/jpm15050175
144	Perspectives on Managing AI Ethics in the Digital Age	2025	Information	Regulatory Challenges	Austrália	Not specified	Narrative Review / Perspective	10.3390/info16040318
145	Perspectives on Using Artificial Intelligence to Derive Social Determinants of Health Data From Medical Records in Canada: Large Multijurisdictional Qualitative Study	2025	Journal of Medical Internet Research	Patient Autonomy	North America	Internal / General Medicine	Qualitative Study	10.2196/52244
146	Privacy, ethics, transparency, and accountability in AI systems for wearable devices	2025	Frontiers in Digital Health	Regulatory Challenges	Europe	Not specified	Narrative Review / Perspective	10.3389/fdgth.2025.1431246
147	Proposal for Responsible Use of Generative Artificial Intelligence in Medical Practice	2025	Revista de Neurología	Regulatory Challenges	Europe	Not specified	Journal Article	10.31083/RN37503
148	Proposing a Principle-Based Approach for Teaching AI Ethics in Medical Education	2024	JMIR Medical Education	Patient Autonomy	Europe	Public health	Narrative Review / Perspective	10.2196/55368
149	Qualitative Evaluation of an Artificial Intelligence–Based Clinical Decision Support System to Guide Rhythm Management of Atrial Fibrillation: Survey Study	2022	JMIR Formative Research	Transparency and Explainability	North America	Cardiology	Qualitative Study	10.2196/36443
150	Reclaiming XAI as an Innovation in Healthcare: Bridging Rule-Based Systems	2025	Algorithms	Transparency and Explainability	Ásia	Not specified	Systematic Review	10.3390/a18090586
151	Recruitment Challenges in Spinal Cord Stimulation Trial for Motor Recovery in Patients with Chronic Complete Spinal Cord Injury	2025	Journal of Clinical Medicine	Transparency and Explainability	Asia	Neurology	Experimental / Trial	10.3390/jcm14113925
152	Responsible AI practice and AI education are central to AI implementation: a rapid review for all medical imaging professionals in Europe	2023	BJR|Open	Beneficence vs Non-Maleficence	Europe	Radiology / Imaging	Journal Article	10.1259/bjro.20230033
153	Responsible artificial intelligence for addressing equity in oral healthcare	2024	Frontiers in Oral Health	Transparency and Explainability	North America	Dentistry	Journal Article	10.3389/froh.2024.1408867
154	Revolutionizing Oncology Through AI: Addressing Cancer Disparities by Improving Screening, Treatment, and Survival Outcomes via Integration of Social Determinants of Health	2025	Cancers	Justice and Equity	North America	Public health	Experimental / Trial	10.3390/cancers17172866
155	Role of Ethics in Developing AI-Based Applications in Medicine: Insights From Expert Interviews and Discussion of Implications	2024	JMIR AI	Regulatory Challenges	Europe	Not specified	Qualitative Study	10.2196/51204

ID	Title	Year	Journal	Main Ethical Theme	Region	Clinical specialty	Article type	DOI
156	Scoping Review: Legal and Ethical Principles of Artificial Intelligence in Public Health	2023	Studies in Health Technology and Informatics	Justice and Equity	Asia	Public health	CHAP	10.3233/SHTI230579
157	Septic Shock in Hematological Malignancies: Role of Artificial Intelligence in Predicting Outcomes	2025	Current Oncology	Responsibility and Accountability	Europe	Public health	Journal Article	10.3390/curroncol32080450
158	Shaping the Future of Healthcare: Ethical Clinical Challenges and Pathways to Trustworthy AI	2025	Journal of Clinical Medicine	Responsibility and Accountability	Europe	Not specified	Narrative Review / Perspective	10.3390/jcm14051605
159	Should Artificial Intelligence-Based Patient Preference Predictors Be Used for Incapacitated Patients? A Scoping Review of Reasons to Facilitate Medico-Legal Considerations	2025	Healthcare	Justice and Equity	Europa	Not specified	Scoping Review	10.3390/healthcare13060590
160	Socio-ethical challenges and opportunities for advancing diversity, equity, and inclusion in digital medicine	2024	DIGITAL HEALTH	Justice and Equity	Europe	Not specified	Narrative Review / Perspective	10.1177/20552076241277705
161	Stage-Wise IoT Solutions for Alzheimer's Disease: A Systematic Review of Detection, Monitoring, and Assistive Technologies	2025	Sensors	Justice and Equity	Europe	Neurology	Systematic Review	10.3390/s25175252
162	Systematic Review of the Application of Artificial Intelligence in Healthcare and Nursing Care	2024	Malaysian Journal of Medical Sciences	Regulatory Challenges	Asia	Nursing	Systematic Review	10.21315/mjms2024.31.5.9
163	The ethical adoption of artificial intelligence in radiology	2020	BJR|Open	Regulatory Challenges	Europe	Radiology / Imaging	Narrative Review / Perspective	10.1259/bjro.20190020
164	The ethical considerations of integrating artificial intelligence into surgery: a review	2025	Interdisciplinary CardioVascular and Thoracic Surgery	Patient Autonomy	Europe	Surgery	Journal Article	10.1093/icvts/ivae192
165	The ethics of ChatGPT in medicine and healthcare: a systematic review on Large Language Models (LLMs)	2024	npj Digital Medicine	Justice and Equity	Europe	Not specified	Systematic Review	10.1038/s41746-024-01157-x
166	The ethics of data mining in healthcare: challenges, frameworks, and future directions	2025	BioData Mining	Transparency and Explainability	Africa	Surgery	Journal Article	10.1186/s13040-025-00461-w
167	The ethics of non-explainable artificial intelligence: an overview for clinical nurses	2025	British Journal of Nursing	Transparency and Explainability	Europe	Nursing	Journal Article	10.12968/bjon.2024.0394

ID	Title	Year	Journal	Main Ethical Theme	Region	Clinical specialty	Article type	DOI
168	The Evolution of Artificial Intelligence in Medical Imaging: From Computer Science to Machine and Deep Learning	2024	Cancers	Transparency and Explainability	Europe	Radiology / Imaging	Experimental / Trial	10.3390/cancers16213702
169	The Impact of Artificial Intelligence on Lung Cancer Diagnosis and Personalized Treatment	2025	International Journal of Molecular Sciences	Regulatory Challenges	Europe	Oncology	Journal Article	10.3390/ijms26178472
170	The METRIC-framework for assessing data quality for trustworthy AI in medicine: a systematic review	2024	npj Digital Medicine	Transparency and Explainability	Europe	Not specified	Systematic Review	10.1038/s41746-024-01196-4
171	The Nocebo Effect: A Bias in Clinical Practice—An Ethical Approach	2024	American Journal of Therapeutics	Patient Autonomy	Europe	Cardiology	Journal Article	10.1097/MJT.0000000000001730
172	The PIEE Cycle: A Structured Framework for Red Teaming Large Language Models in Clinical Decision-Making	2025	Bioengineering	Beneficence vs Non-Maleficence	North America	Surgery	Narrative Review / Perspective	10.3390/bioengineering12070706
173	The Potential of Artificial Intelligence in the Diagnosis and Prognosis of Sepsis: A Narrative Review	2025	Diagnostics	Transparency and Explainability	Europe	Oncology	Narrative Review / Perspective	10.3390/diagnostics15172169
174	The Promise and Perils of Artificial Intelligence in Advancing Participatory Science and Health Equity in Public Health	2025	JMIR Public Health and Surveillance	Justice and Equity	North America	Public health	Journal Article	10.2196/65699
175	The Promise of Artificial Intelligence in Digestive Healthcare and the Bioethics Challenges It Presents	2023	Medicina	Privacy and Data Protection	Europe	Radiology / Imaging	Journal Article	10.3390/medicina59040790
176	The risks of artificial intelligence: A narrative review and ethical reflection from an Oral Medicine group	2025	Oral Diseases	Responsibility and Accountability	Europe	Dentistry	Narrative Review / Perspective	10.1111/odi.15100
177	The Role of AI-Based Chatbots in Public Health Emergencies: A Narrative Review	2025	Future Internet	Impact on the Medical Profession	Europe	Public health	Narrative Review / Perspective	10.3390/fi17040145
178	Toward Decentralized Intelligence: A Systematic Literature Review of Blockchain-Enabled AI Systems	2025	Information	Responsibility and Accountability	North America	Not specified	Journal Article	10.3390/info16090765
179	Toward Fairness, Accountability, Transparency, and Ethics in AI for Social Media and Health Care: Scoping Review	2024	JMIR Medical Informatics	Transparency and Explainability	North America	Not specified	Scoping Review	10.2196/50048
180	Towards Navigating Ethical Challenges in AI-Driven Healthcare Ad Moderation	2025	Computers	Responsibility and Accountability	North America	Internal / General Medicine	Narrative Review / Perspective	10.3390/computers14090380

ID	Title	Year	Journal	Main Ethical Theme	Region	Clinical specialty	Article type	DOI
181	Towards Transparent Healthcare: Advancing Local Explanation Methods in Explainable Artificial Intelligence	2024	Bioengineering	Transparency and Explainability	Europe	Not specified	Journal Article	10.3390/bioengineering11040369
182	Transforming Cancer Care: A Narrative Review on Leveraging Artificial Intelligence to Advance Immunotherapy in Underserved Communities	2025	Journal of Clinical Medicine	Justice and Equity	North America	Oncology	Narrative Review / Perspective	10.3390/jcm14155346
183	Transforming Speech-Language Pathology with AI: Opportunities, Challenges, and Ethical Guidelines	2025	Healthcare	Transparency and Explainability	Europe	Public health	Journal Article	10.3390/healthcare13192460
184	Transparency of AI in Healthcare as a Multilayered System of Accountabilities: Between Legal Requirements and Technical Limitations	2022	Frontiers in Artificial Intelligence	Transparency and Explainability	Europe	Not specified	Narrative Review / Perspective	10.3389/frai.2022.879603
185	Trust in Artificial Intelligence–Based Clinical Decision Support Systems Among Health Care Workers: Systematic Review	2025	Journal of Medical Internet Research	Transparency and Explainability	Asia	Not specified	Systematic Review	10.2196/69678
186	Trustworthy and ethical AI-enabled cardiovascular care: a rapid review	2024	BMC Medical Informatics and Decision Making	Responsibility and Accountability	North America	Cardiology	Journal Article	10.1186/s12911-024-02653-6
187	Unlocking the Potential of AI in EUS and ERCP: A Narrative Review for Pancreaticobiliary Disease	2025	Cancers	Justice and Equity	Europe	Radiology / Imaging	Narrative Review / Perspective	10.3390/cancers17071132

Supplementary File S2: Python script for weighted keyword relevance scoring (high, medium, low relevance).

```
# RELEVANCE ANALYSIS
# Model: 2 + 1 points (no duplications)
import re
import pandas as pd
from pathlib import Path
# Configurations
INPUT = "input_refworks.txt"
OUTPUT = "Relevance Analysis.xlsx"
# --- Words of High weight (2 pts)
HIGH_WEIGHT = [
   "ethic","bioethic","accountability","responsibility","liability",
   "explainab","interpretab","xai","transparency","black box",
   "autonomy","consent","informed consent","fairness","bias","equity",
   "privacy","gdpr","data protection","trust","governance","oversight"
]
# Words of Medium weight (1 pt)
MEDIUM_WEIGHT = [
   "ai in medicine","clinical ai","healthcare ai",
   "decision support","medical ai"
]
# Compile (substring match)
def to_regex(term):
   term = term.lower().strip()
   return re.compile(re.escape(term), re.IGNORECASE)
PATTERNS_HIGH = {t: to_regex(t) for t in HIGH_WEIGHT}
PATTERNS_MED  = {t: to_regex(t) for t in MEDIUM_WEIGHT}
# Read file RefWorks
txt = Path(INPUT).read_text(encoding="utf-8", errors="ignore")
records = re.split(r"(?=^RT\b)", txt, flags=re.MULTILINE)
records = [r.strip() for r in records if r.strip()]
def parse_record(block):
   data = {}
   for line in block.splitlines():
      line = line.strip()
      if not line:
         continue
      m = re.match(r"^([A-Z0-9]{2,3})\s*-\s*(.*)$", line)
      if not m:
         m = re.match(r"^([A-Z0-9]{2,3})\s+(.*)$", line)
      if m:
         key, val = m.groups()
         data.setdefault(key, []).append(val.strip())
      else:
         if data:
            data[list(data.keys())[-1]][-1] += " " + line
   return {k: " ".join(v) for k, v in data.items()}
parsed = [parse_record(r) for r in records]
# Extract
```

```
rows = []
for d in parsed:
    title = d.get("T1", "").strip()
    if not title:
        continue
    abstract = d.get("AB", "").strip() or d.get("N2", "").strip()
    year = (d.get("YR", "") or d.get("PY", "")).strip()
    doi = (d.get("DO", "") or d.get("DOI", "")).strip()
    rows.append({
        "Title": title,
        "Abstract": abstract,
        "Year": year,
        "DOI": doi
    })
df = pd.DataFrame(rows)
# Score Calculation
scores, hits_all, levels = [], [], []
for _, r in df.iterrows():
    text = f"{r['Title']} {r['Abstract']}".lower()
    # Unique words found
    found_high = [t for t, p in PATTERNS_HIGH.items() if p.search(text)]
    found_med  = [t for t, p in PATTERNS_MED.items()  if p.search(text)]
    score = len(found_high)*2 + len(found_med)*1
    scores.append(score)
    hits_all.append(", ".join(found_high + found_med))
    # Classification
    if score >= 6:
        lvl = "High"
    elif score >= 3:
        lvl = "Medium"
    else:
        lvl = "Low"
    levels.append(lvl)
df["Score"] = scores
df["Hits"] = hits_all
df["Relevance Level"] = levels
#  Summary
summary = (
    df["Relevance Level"]
    .value_counts()
    .rename_axis("Relevance Level")
    .reset_index(name="Count")
)
summary["Percent"] = (summary["Count"]/len(df)*100).round(2)
# Save to Excel
with pd.ExcelWriter(OUTPUT, engine="openpyxl") as writer:
    df.to_excel(writer, sheet_name="General", index=False)
    df[df["Relevance Level"] == "High"].to_excel(writer, sheet_name="High Relevance", index=False)
    summary.to_excel(writer, sheet_name="Global summary", index=False)
)
```

Supplementary File S3: Python script for classification of high-relevance studies into eight ethical categories.

```
#  Exclusive Classification in the 8 Ethical Categories
#  Input  : Relevance Analysis.xlsx
#  Output : Analysis_Categories.xlsx
import re
import pandas as pd
from pathlib import Path
IN_FILE  = "Relevance Analysis.xls"
IN_SHEET = "High Relevance"
OUT_FILE = "Category.xlsx"
# Categories e keywords (roots)
CATEGORIES = [
    ("Transparency and Explainability",
     ["transparency","explain","interpretab","xai","black box","interpretability","clarity","understand"]),
    ("Responsibility and Accountability",
     ["responsib","accountab","liabil","duty","oversight","govern","oblig","answerab"]),
    ("Regulatory Challenges",
     ["regulat","legal","law","complian","standard","policy","framework"]),
    ("Justice and Fairness",
     ["fair","bias","equit","discriminat","inclus","justic"]),
    ("Patient Autonomy",
     ["autonom","consent","informed consent"," patient decision","choice","self-determin"]),
    ("Beneficence vs Non-Maleficence",
     ["benefic","non-malef","harm"," patient safety","well-being","risk"]),
    ("Privacy and Data Protection",
     ["privac","gdpr","data protect","confident","anonym","sensitive data"]),
    ("Impact on the Medical Profession",
     ["job","role","clinician","workload","substitut","human oversight","skill","competenc"]),
]
def classify_exclusive(text: str) -> str:
    """Returns the category with the highest number of matches (substring), tied by the above order."""
    t = (text or "").lower()
    best_cat = "No match"
    best_score = 0
    for cat, kws in CATEGORIES:
        # single occurrence per keyword (non-duplicative)
        score = sum(1 for kw in kws if re.search(re.escape(kw), t))
        if score > best_score:
            best_cat = cat
            best_score = score
    return best_cat
def main():
    # Read entry
    df = pd.read_excel(IN_FILE, sheet_name=IN_SHEET)
    if "Title" not in df.columns or "Abstract" not in df.columns:
        raise ValueError("The “Relevance Analysis” sheet should contain “Title” and “Abstract” columns.")
    # Classify
    df["Main Ethics Category"] = df.apply(
        lambda r: classify_exclusive(f"{r['Title']} {r['Abstract']}"), axis=1
    )
```

```
# Summary
summary = (
    df["Main Ethics Category"]
    .value_counts()
    .rename_axis("Main Ethics Category")
    .reset_index(name="Number of items")
)
# Save Excel
with pd.ExcelWriter(OUT_FILE, engine="openpyxl") as writer:
    df.to_excel(writer, sheet_name="Analysis_Categories", index=False)
    summary.to_excel(writer, sheet_name="Summary Categories", index=False)
```

Supplementary File S4: Keyword list by ethical category

Categories	Keywords
Transparency and Explainability	transparency, explainability, interpretab, xai, black box, interpretability, clarity, understand
Regulatory Challenges	regulation, legal, law, compliance, standard, policy, framework
Responsibility and Accountability	responsib, accountab, liability, duty, oversight, governance, obligation, answerab
Justice and Equity	fairness, bias, equity, discrimination, inclusivity, justice
Patient Autonomy	autonomy, consent, informed consent, patient decision, choice, self-determination
Beneficence vs Non-Maleficence	beneficence, non-maleficence, harm, patient safety, well-being, risk
Privacy and Data Protection	privacy, gdpr, data protection, confidentiality, anonymization, sensitive data
Impact on the Medical Profession	job, role, clinician, workload, substitution, human oversight, skill, competence

Supplementary File S5: Grey Literature

1. U.S. Food and Drug Administration (FDA); Health Canada; Medicines and Healthcare Products Regulatory Agency (MHRA). *Good Machine Learning Practice for Medical Device Development: Guiding Principles*; FDA: Silver Spring, MD, USA, **2021**.
2. European Medicines Agency (EMA). *Reflection Paper on the Use of Artificial Intelligence (AI) in the Medicinal Product Lifecycle*; EMA: Amsterdam, The Netherlands, **2024.**
3. World Health Organization (WHO). *Guidance on Ethics and Governance of Artificial Intelligence for Health*; WHO: Geneva, Switzerland, **2021**.
4. European Union. *Artificial Intelligence Act*; EU: Brussels, Belgium, **2024**.
5. Organisation for Economic Co-operation and Development (OECD). *OECD Principles on Artificial Intelligence*; OECD: Paris, France, **2019**.
6. United Nations Educational, Scientific and Cultural Organization (UNESCO). *Recommendation on the Ethics of Artificial Intelligence*; UNESCO: Paris, France, **2021**.
7. United Nations Educational, Scientific and Cultural Organization (UNESCO). *Guidelines on Ethical and Inclusive AI for Health*; UNESCO: Paris, France, **2023**.
8. Medicines and Healthcare Products Regulatory Agency (MHRA). *Software and AI as a Medical Device Change Programme*; MHRA: London, UK, **2024**.

9. Health Canada. *Proposed Regulatory Framework for Machine Learning-Enabled Medical Devices*; Health Canada: Ottawa, ON, Canada, **2023**.
10. U.S. Department of Health and Human Services (HHS). *Trustworthy AI Playbook for Health Systems*; HHS: Washington, DC, USA, **2021**.
11. I International Organization for Standardization; International Electrotechnical Commission. *Artificial Intelligence—Risk Management—ISO/IEC 42001*; ISO: Geneva, Switzerland, **2023**.
12. Council of Europe. *Guidelines on Artificial Intelligence, Human Rights, Democracy and the Rule of Law*; Council of Europe: Strasbourg, France, **2022**.
